# Unraveling the anti-inflammatory effects of Mediterranean diet in patients with cancer remission

**DOI:** 10.3389/fimmu.2025.1666611

**Published:** 2025-12-02

**Authors:** Michele Francesco Di Tolla, Michele Libutti, Giovanna D’Onofrio, Alessandra Riccio, Serena Cabaro, Michele Longo, Alessia Parascandolo, Giusy Ferraro, Elia Formisano, Vittoria D’Esposito, Pietro Formisano

**Affiliations:** 1 Department of Translational Medical Sciences, University of Naples “Federico II”, Naples, Italy; 2 Oncology Department, Azienda Sanitaria Locale Napoli 3 Sud, Naples, Italy; 3 Department of Cognitive Neurosciences, Maastricht University, Maastricht, Netherlands; 4 Institute of Endotypes in Oncology, Metabolism and Immunology “G. Salvatore” – National Research Council (IEOMI-CNR), Naples, Italy

**Keywords:** LGCI, cytokines, mediterranean diet, cancer, lifestyle

## Abstract

**Introduction:**

Cancer survivors display impaired quality of life and increased risk to develop cardiometabolic comorbidities. Low-grade chronic inflammation (LGCI) plays a crucial role in cancer progression and in cardiometabolic diseases. Although the Mediterranean Diet is widely recognized for its beneficial effects on body composition and systemic inflammation, direct evidence of its impact on cancer survivors remains limited. This study aimed to explore the associations between inflammatory biomarkers, metabolic status, and Mediterranean diet adherence, evaluating the effectiveness of a personalized dietary intervention.

**Methods:**

A total of 132 patients with cancer remission were enrolled; anamnestic, anthropometric, bioimpedential, clinical, and nutritional data were collected. Serum concentrations of cytokines, chemokines, growth factors, and metabolic markers were measured at baseline and at a six-month follow-up.

**Results:**

Baseline analysis revealed distinct clusters linking inflammatory status, clinical variables, and metabolic profiles, confirming associations between systemic inflammation and body composition features, without clear separation among cancer types. After dietary intervention, a significant reduction in specific inflammatory and metabolic biomarkers was observed, with distinct effects depending on tumor type. For instance, Leptin and Insulin levels decreased, particularly in Breast cancer patients, whereas Colorectal cancer patients exhibited a reduction in pro-inflammatory cytokines such as IL-1β, IL-6, IL-8, and TNF-α, even in the absence of weight loss and bioimpedential feature changes. Retrospective analysis further highlighted that nutritionally modified molecules were associated with metabolic and inflammatory risk factors at baseline.

**Discussion:**

Despite the lack of a control group and the high attrition rate may represent limitations for this observational pilot study, we have provided evidence that nutritional intervention could be a promising complementary strategy in oncological management by modulating key inflammatory and metabolic pathways involved in tumor pathophysiology and comorbidities.

## 1 Introduction

Cancer is one of the leading causes of morbidity and mortality worldwide. According to the Global Cancer Observatory (GLOBOCAN), over 20 million new cases and 9.7 million associated deaths were reported in 2022 (1). The increase in cases is attributed to an aging population and behavioral and environmental factors such as poor diet, sedentary lifestyle, and pollution. Beyond its direct impact on survival, cancer significantly impairs the quality of life of patients, which may ease the incidence of comorbidities like cardiovascular diseases and metabolic disorders (2, 3).

In fact, despite significant advances in cancer treatment, patients in remission may also face metabolic comorbidities and chronic inflammatory states. These factors not only impact on the quality of life but can also increase the risk of recurrence and other complications (4, 5). Low-Grade Chronic Inflammation (LGCI), often referred to as “metabolic inflammation”, plays a pivotal role in linking metabolic disorders with cancer progression and recurrence (6, 7). LGCI is highly influenced by age, BMI, smoke, physical activity and dietary habits. In general population, these factors are associated with changes in specific circulating cytokines, including IL-1β, IL-6, IL-8, and TNF-α (8). However, these cytokines also possess a well-described role in cancer and tumor microenvironment alteration, promoting cell proliferation and angiogenesis (9). Thus, adopting targeted lifestyle interventions, including specific dietary modifications, represents a promising strategy to improve clinical and metabolic profile of patients with cancer (10).

In this context, the effectiveness of the Mediterranean diet in promoting healthy body composition, improving metabolic parameters, and reducing the systemic inflammation has been largely demonstrated (11, 12). However, identification of molecular markers is still needed to trace lifestyle and dietary habit changes, and to evaluate their impact on metabolic and inflammatory patterns in patients with “remissible” cancers.

This study aimed to analyze the effects of a dietary intervention based on the Mediterranean model on a cohort of patients in cancer remission. Previously, we described how, in healthy individuals, lifestyle factors and dietary habits impact on circulating levels of cytokines and chemokines, defining a molecular signature of LGCI (8). Cytokines and chemokines play a crucial role in cancer onset and progression; however, the impact of lifestyle, oncological status and comorbidities on their circulating levels in cohorts of patients with cancer remission has never been explored. We have used an integrative approach combining nutritional monitoring with detailed analysis of molecular biomarkers, including cytokines, chemokines, and metabolic markers to identify significant associations between clinical, nutritional, and molecular variables, and to provide an integrated view of the underlying dynamics. Understanding the interactions between diet, inflammatory, and metabolic state can provide new perspectives for preventive and therapeutic interventions (13–15).

## 2 Materials and methods

### 2.1 Population enrollment and characterization

In this observational study, 166 consecutive patients with remissive cancer were recruited at the Local Health Unit of Naples 3 South, Naples, Italy. Inclusion criteria were:

- Partial or complete cancer remission;
- Age ≥ 18 years;
- Understanding and acceptance of informed consent.

Exclusion criteria were:

- Inability to eat per os;
- Proven difficulty in ambulating;
- Suffering from severe side effects caused by antineoplastic therapies;
- Refused to give informed consent.

All enrolled volunteers underwent detailed and bioimpedance phenotyping, including measurement of height, weight, waist and hip circumferences. Height was measured with a wall-mounted stadiometer GIMA™ 27335, while waist and hip circumferences with a SECA™ 201 non-stretchable measuring tape. Bioimpedance parameters were assessed by TANITA ™ MC-780U multifrequency segmental body composition analyzer, and included Weight, Fat Mass (FM), Fat-Free Mass (FFM), Basal Metabolic Rate (BMR), Total Body (TBW) and Extra-Cellular Water (ECW), Visceral Adipose Tissue (VAT), Bone Weight, and Phase Angle (°). Body Mass Index (BMI) was calculated as a ratio of body weight/height^2^ (kg/m^2^).

Clinical and Pathological data were collected at time of enrolment (T0) and after a 6-month follow-up (T1). Data included cancer type, presence of metastasis, cancer recurrence, and eventual ongoing chemotherapy/treatment status, as well as eventual comorbidities, like Metabolic Syndrome (METS), Type-2 Diabetes (T2D), cardiovascular diseases (CVD), Non-Alcoholic Fatty Liver Disease (NAFLD), Hyperlipaemia (HL), and Anemia (A). Moreover, a detailed questionnaire about anamnestic and behavioral data, such as smoking habit, physical activity, and weekly food frequency, was administered to each volunteer (as described below). Cytometric blood counts, biochemical analyses, and tumor-marker screenings were collected as well.

A serum sample from all donors was obtained at T0 and T1, and stored at -20°C.

Investigations were carried out following the rules of the Declaration of Helsinki of 1975, revised in 2013. Informed consent was obtained from every volunteer before the procedure. The protocol was approved by the ethical committee of the University of Naples.

### 2.2 Nutrition data collection

The assessment of volunteers’ nutrition habits was based on the procedure described in D’Esposito et al. (2022) (8). Briefly, a food frequency questionnaire (FFQ) was de visu administered by two expert nutritionists. The FFQ involved information about the last 7 days of food frequency and amount and was based on the Italian EPIC study (16, 17). Consequently, a list of 12 food groups (namely Cereals, Milk, Seafood, Crustaceans, Meat, Poultry, Processed Meats, Vegetables, Fruits, Cakes, Pizza, and Beverage) was developed in line with the local food availability, cultural specificity and dietary habits. Food frequency type with portion size was estimated by means of pictures. Pictures showing different portion sizes – arranged by increased amount – followed the question on frequency and corresponded to a specific portion in grams. Questionnaires were finally digitalized, and data related to the weekly total consumption of each food item were gathered and analyzed.

### 2.3 Dietary intervention

The dietary intervention consisted of a qualitative dietary change characterized by low glycemic and insulinemic indexes, with the Mediterranean model as a blueprint. The intervention objectives were the promotion of a healthy diet, the mitigation of antineoplastic therapy side effects, the improvement of patients’ metabolic derangement, inflammatory status, and body composition.

To achieve these goals, all participants were provided with the Italian Dietary Guidelines for Healthy Eating, released by the Research Center for Food and Nutrition of CREA (Council for Agricultural Research and Economics) (18, 19). These guidelines, grounded in the Mediterranean dietary model, take into account the recommendations of World Cancer Research Fund (WCRF) (20), and encourage to:

- Include fiber-rich foods such as whole grains or their derivatives, legumes, and non-starchy vegetables;
- Reduce the intake of foods with a high glycemic index (e.g., refined flour, simple sugars) and high insulinemic index (e.g., cow’s milk);
- Limit foods rich in saturated fats (e.g. red and processed meats, dairy products) and avoid trans-fat sources (e.g., margarine and baked goods);
- Decrease the consumption of animal-derived foods, favoring legumes as a source of plant-based proteins. Among animal-derived foods, prioritize small oily fish rich in Omega-3 fatty acids;
- Use extra-virgin olive oil as the primary fat for cooking and seasoning;
- Flavor dishes with spices and herbs to minimize salt consumption.

In detail, the Mediterranean dietary protocol administered to patients consisted of three main meals (breakfast, lunch, and dinner) and two snacks (mid-morning and mid-afternoon). Breakfast included fermented milk products (e.g., yogurt) and baked goods or cereals (flakes or puffed), preferably whole-grain or based on ancient grains. Lunch and dinner were structured around a generous portion of fresh or cooked seasonal, locally sourced vegetables; a source of complex carbohydrates such as whole grains (e.g., pasta, bread, or grain kernels such as rice, spelt, and barley) or potatoes (twice per week); and a moderate portion of proteins, with a preference for legumes and fish (at least three times per week), white meat (2–3 times per week), ricotta or aged cheeses such as Parmigiano Reggiano™, and eggs (twice per week), while limiting red meat and processed meats to once per week. Extra-virgin olive oil was recommended as the main condiment for both lunch and dinner. For snacks, options included fresh fruit, fermented milk products (yogurt), nuts, and oilseeds. In addition, participants were allowed one glass of red wine if desired, and a dessert once per week.

These recommendations allowed to standardize the dietary approach even for patients with metabolic comorbidities (e.g. T2D, Hyperlipaemia, NAFLD).

To ensure greater adherence to the prescribed dietary plan, periodic nutritional follow-up visits were conducted approximately every 30 days, allowing for the evaluation of anthropometric and bioimpedance parameters. During these sessions, adherence to the Mediterranean diet model was monitored through an intervention-modulated FFQ, and nutritional counseling was provided to guide patients through their dietary transition and behavioral change, as well as tracking the overall quality-of-life and clinical follow-up.

### 2.4 Determination of cytokines, chemokines, growth factors and metabolic makers

Serum samples at T0 and T1 were screened for the concentration of interleukin (IL)-1β, IL-1ra, IL-2, IL-4, IL-5, IL-6, IL-7, IL-8, IL-9, IL-10, IL-12 (p70), IL-13, IL-15, IL-17A, Eotaxin, basic Fibroblast Growth Factor (bFGF), Granulocyte-Colony Stimulating Factor (G-CSF), Granulocyte and Macrophage-Colony Stimulating Factor (GM-CSF), Interferon-γ (IFN-γ), Interferon-γ inducible Protein-10 (IP-10), Monocyte chemoattractant Protein-1 (MCP-1), Macrophage Inflammatory Protein-1 alpha (MIP-1α), MIP-1β, C-C motif chemokine ligand 5/Regulated on Activation, Normal T-cell Expressed and Secreted (CCL5/RANTES), Tumor Necrosis Factor-α (TNF-α), Platelet-Derived Growth Factor-bb (PDGF), and Vascular Endothelial Growth Factor (VEGF), using the Bio-Plex^®^ Multiplex Human Cytokine, Chemokine, and Growth Factor Kit (cat. number M500KCAF0Y, Bio-Rad, Hercules, Ca, USA), as previously described (8, 21, 22).

Additionally, a second array of metabolic markers, including C-Peptide, Ghrelin, Gastric Inhibitory Peptide (GIP), Glucagon-Like Peptide-1 (GLP-1), Glucagon, Insulin, Leptin, total Plasminogen Activator Inhibitor-1 (PAI-1), Resistin, and Visfatin, was screened using the Bio-Plex Pro^®^ Human Diabetes 10-plex Assay (cat. number 171A7001M, Bio-Rad, Hercules, Ca, USA), as previously described (23–25). Both magnetic bead-based assays were performed on a Bio-Plex 200 analyzer with the Bio-Rad Bio-Plex Manager software (Bio-Rad, Hercules, CA, United States).

High sensitivity C-Reactive Protein (CRP, cat. number L2KCR2, Immulite, USA) assay was performed using the IMMULITE^®^ 2000 Analyzer (DPC, Los Angeles, Ca, USA), according to the manufacturer’s protocol. High Molecular Weight Adiponectin (cat. number 296752, Fujirebio, Tokyo, Japan) CLIA assay was performed using the Lumipulse^®^ G600II analyzer (Fujirebio, Tokyo, Japan).

### 2.5 Statistical analysis

Statistical Analysis was performed using both GraphPad^®^ Prism 8.4.2 software (GraphPad Software Inc, La Jolla, Ca, USA) and the R version 4.1.2 programming language (R Foundation for Statistical Computing, Vienna, Austria) through the RStudio^®^ Integrated Development Environment (Posit PBC, Boston, Ma, USA).

Categorical Variables were described as the number of occurrences and relative frequency as percentage and compared using Fisher’s exact test. Continuous variables were described as either mean ± standard deviation or median and interquartile range (IQR, 25th to 75th percentile). Shapiro-Wilk normality test was used to evaluate whether the continuous data were normally distributed.

Correlations among two continuous variables were reported and analyzed with either Pearson’s r correlation coefficient or Spearman’s rho rank correlation coefficient, and their respective 95% confidence intervals (95% CI). for two-group comparisons, either Welch’s two tailed t-test for independent samples (for parametric data) or Mann Whitney U-test (for non-parametric data) were used. Multiple comparisons among more than two groups were assessed using either ANOVA test with Tukey’s *post hoc* correction (for parametric data) or Kruskal-Wallis test with Dunn’s *post hoc* correction (for non-parametric data). Intra-patient comparisons of variables between T0 and T1 were reported as mean variation (Δ), with 95% CI, and assessed through either paired t-test or paired Wilcoxon test, along with Cohen’s d evaluation and relative 95% CI for effect size assessment. Outliers were detected and accordingly removed with the ROUT (Robust regression and Outlier removal) method of regression. Briefly, a robust nonlinear regression was used to fit a curve not influenced by outliers; then, the residuals of the robust fit analyzed to identify any outliers. This step used an outlier test adapted from the False Discovery Rate approach of testing for multiple comparisons; finally, outliers were removed and a least-squares regression on the remaining data was performed.

High dimensional data visualization algorithms, such as t-distributed stochastic neighbor embedding (t-SNE) and Uniform Manifold Approximation and Projection (UMAP), were used for visualizing the high dimensionality data on two-dimensional projections. After empirical fine-tuning, t-SNE was used with a perplexity factor equal to 30, while UMAP parameter for neighbors was set to 15. Moreover, Multiple Factor Analysis (MFA) was used to both analyze a dimensionality reduction with a proper variable grouping, the single-variable impact on the cumulative overall variance, and the tumor-relative clustering. All analyses were performed with a fixed random seed (04031960) to ensure reproducibility.

To investigate the collinearity effects among risk factors and food groups, data were analyzed with a multivariable linear regression analysis. Generated models were analyzed with an ANOVA test to evaluate the goodness of fit. Regression coefficients were reported as an estimate and their 95% CI.

All p-values <0.05 were considered statistically significant.

## 3 Results

### 3.1 Biometrical and clinical features

166 consecutive patients with cancer in remission were recruited. Of these, 64 had Breast Cancer (BC), 52 had Colorectal Cancer (CC), and 16 had Prostate Cancer (PC). 34 of them had poorly represented tumors (e.g. Endometrial Cancer, n: 6; Bladder Cancer, n: 5; Kidney Cancer, n: 4) and were not included in further analyses.

At time of enrolment (T0), BC, CC, and PC patients displayed a well distinct characterization, with the BC groups being the youngest (mean age: 55.88 ± 11.06 years) but with the highest BMI (median: 29.56 Kg/m^2^, IQR 26.81 to 33.98 Kg/m^2^). These differences were maintained after stratifying age in 10-year range subgroups and BMI in Normal Weight, Overweight, Obese. Apart from slightly lower alcohol exposure for BC patients, no differences in behavioral, blood count and biochemical data were noted (**Table 1**, **Supplementary Table S1**).

**Table 1:** Clinical phenotyping of the enrolled population.

Parameters (Unit)	Total (N: 132)	BC (N: 64, 48.48%)	CC (N: 52, 39.39%)	PC (N: 16, 12.12%)	Overall p-value
Anagraphic features					
Sex (Females, %)	84 (63.64%)	63 (98%)	21 (40.38%)	0 (0%)	**<0.001**
Age (Years)	60.95 ± 11.06	55.88 ± 11.06 ***^b,c^***	63.37 ± 8.53 ***^a,c^***	73.32 ± 7.73 ***^a,b^***	**<0.001**
<50 (%) 50-59 (%) 60-69 (%) ≥70 (%)	24 (18.18%) 39 (29.55%) 39 (29.55%) 30 (22.72%)	20 (31.25%) 23 (35.94%) 15 (23.44%) 6 (9.38%)	4 (7.69%) 15 (28.85%) 20 (38.46%) 13 (25%)	0 (0%) 1 (6.25%) 4 (25%) 11 (68.75%)	**<0.001**
Height (m)	1.64 [1.57; 1.70]	1.61 [1.56; 1.65] ***^b,c^***	1.66 [1.59; 1.73] ***^a,c^***	1.7 [1.64; 1.75] ***^a,b^***	**<0.001**
Weight (Kg)	78.7 [67.77; 87.5]	78.9 [68.47; 88.4]	77.75 [63.08; 84.1]	81.5 [68.25; 88.95]	0.628
Phys. Act. (%)	54 (40.9%)	21 (32.81%)	24 (46.15%)	9 (56.25%)	0.143
Smokers (%)	28 (21.21%)	11 (17.19%)	12 (23.07%)	5 (31.25%)	0.429
Alcohol (%)	44 (33.3%)	14 (21.88%) ***^b^***	23 (44.23%) ***^a^***	7 (43.75%)	**0.025**
Sleep Time (hrs)	6.26 ± 1.47	6.16 ± 1.6	6.31 ± 1.35	6.5 ± 1.37	0.739
Sleep Quality (%) Low (%) Medium (%) High (%)	56 (42.42%) 35 (26.52%) 41 (31.06%)	29 (45.32%) 18 (28.12%) 17 (26.56%)	19 (36.54%) 14 (26.92%) 19 (36.54%)	8 (50%) 3 (18.75%) 5 (31.25%)	0.745
Bioimpedential features					
BMI (Kg/m^2^)	28.65 [25.41; 32.51]	29.56 [26.81; 33.98] ***^b,c^***	27.13 [24.37; 31.26] ***^a^***	26.76 [23.62; 30.71] ***^a^***	**0.012**
NW (%) OW (%) OB (%)	32 (24.25%) 48 (36.36%) 52 (39.39%)	9 (14.06%) 25 (39.06%) 30 (46.88%)	16 (30.77%) 20 (38.46%) 16 (30.77%)	7 (43.75%) 3 (18.75%) 6 (37.50%)	**0.046**
Waist (cm)	103.5 [95; 112.5]	103 [93.25; 112]	100.5 [97; 112.75]	106 [98.5; 112.5]	0.859
Hip (cm)	106 [101.25; 117.25]	111.5 [104; 119] ***^b,c^***	104 [99; 115] ***^a^***	104.5 [98.5; 106.38] ***^a^***	**0.018**
WHR	0.95 [0.89; 1.00]	0.9 [0.87; 0.96] ***^b,c^***	0.98 [0.94; 1.02] ***^a^***	1.01 [0.95; 1.03] ***^a^***	**<0.001**
FM (Kg)	24.3 [18.75; 32.85]	26.1 [22.3; 36.8] ***^b,c^***	19.95 [15.3; 28.28] ***^a^***	20.95 [16.97; 27.65] ***^a^***	**<0.001**
FFM (Kg)	51.3 [46.2; 56.2]	49.3 [45.1; 53] ***^b,c^***	53.4 [46.58; 58.83] ***^a^***	56.6 [52; 64.27] ***^a^***	**<0.001**
BMR (kcal)	1521 [1370; 1658]	1474 [1358; 1607] ***^b,c^***	1541 [1406; 1727] ***^a^***	1636 [1467; 1847] ***^a^***	**0.024**
TBW (L)	35 [31.7; 40.1]	33 [30.4; 35.6] ***^b,c^***	37.35 [32.73; 42.38] ***^a,c^***	42.15 [37.1; 45.2] ***^a,b^***	**<0.001**
ECW (L)	16.7 [14.85; 18.35]	16.1 [14.4; 17.8] ***^b,c^***	17.2 [15.83; 19] ***^a^***	18.55 [16.8; 20.08] ***^a^***	**0.002**
VAT (Kg)	10 [7; 12]	8 [7; 11] ***^b,c^***	11 [7.25; 13] ***^a,c^***	12.5 [11; 15.25] ***^a,b^***	<**0.001**
Bone (Kg)	2.6 [2.3; 2.8]	2.5 [2.3; 2.7] ***^b,c^***	2.7 [2.4; 2.95] ***^a,c^***	2.8 [2.58; 3.23] ***^a,b^***	**0.002**
Phase (°)	5.4 [5; 5.93]	5.4 [5.1; 5.8]	5.4 [4.95; 6.15]	5.35 [4.95; 5.5]	0.377
Clinical features					
Metastasis (%)	19 (14.39%)	7 (10.94%)	12 (23.08%) ***^c^***	0 (0%) ***^b^***	**0.039**
Chemotherapy (%)	47 (35.61%)	33 (51.56%) ***^b,c^***	12 (23.08%) ***^a^***	2 (12.5%) ***^a^***	**<0.001**
METS (%)	14 (10.61%)	5 (7.81%)	6 (11.53%)	3 (18.75%)	0.429
T2D (%)	20 (15.15%)	6 (9.38%)	11 (21.15%)	3 (18.75%)	0.194
CVD (%)	66 (50%)	29 (45.31%)	25 (48.07%)	12 (75%)	0.098
NAFLD (%)	33 (25%)	17 (26.56%)	12 (23.08%)	4 (25%)	0.911
Hyperlipaemia (%)	59 (44.7%)	30 (46.88%)	19 (36.53%)	10 (62.5%)	0.167
Anemia (%)	7 (5.3%)	4 (6.25%)	2 (3.8%)	1 (6.25%)	0.834

Age is expressed as mean ± standard deviation. Categorical variables are expressed as count (percentage). All other data are reported as median and range [IQR]. p-values in bold are considered statistically significant (p-value <0.05).

Phys Act: Physical Activity, BMI: Body Mass Index; NW: Normal Weight; OW: Overweight; OB: Obese; WHR: Waist-to-Hip ratio; FM: Fat Mass; FFM: Fat-Free Mass; BMR: Basal Metabolic Rate; TBW: Total Body Water; ECW: Extracellular Water; VAT: Visceral Adipose Tissue; METS: Metabolic Syndrome; T2D: Type-2 Diabetes; CVD: Cardiovascular Disease; NAFLD: Non-Alcoholic Fatty Liver Disease. a denotes a significant difference versus the BC group; b denotes a significant difference versus the CC group; c denotes a significant difference versus the PC group.

Bioimpedance data reflected the marked anagraphical and physical differences among the three groups. Presence of Metastasis was reported mainly for CC patients (23.08%), while chemotherapic treatment for BC patients (51.56%) (**Table 1**).

Dietary information was collected with a weekly food-frequency questionnaire, as described in the “Materials and Methods”, and further analyzed. No gross differences in dietary habits based on food frequency and distribution were noted (**Supplementary Table S2**).

Patients’ serum was screened for the concentration of two panels of molecules: a 27-molecules array of cytokines, chemokines, and growth factors, and a 10-molecules array of metabolic markers. At the same time, circulating levels of C-Reactive Protein (CRP) and Adiponectin were screened. Overall, all factors were detectable in serum specimens (**Table 2**). Screened molecules displayed an overall balanced distribution among the three cancer subgroups, with the exception of a slight increase of IL-12 in PC patients, and a remarkably higher concentration of Leptin in BC patients (**Table 2**).

**Table 2:** Serum concentration of cytokines, chemokines, growth factors, and metabolic markers.

Marker	Total (N: 132)	BC (N: 64, 48.48%)	CC (N: 52, 39.39%)	PC (N: 16, 12.12%)	Overall p-value
Cytokines, chemokines and growth factors					
IL-1β	4.71 [3.74; 7.38]	4.39 [3.74; 6.95]	5.25 [3.96; 13.4]	5.58 [4.77; 7.9]	0.313
IL-1ra	184.9 [134.52; 230.8]	181.49 [121.1; 229.2]	184.91 [149.6; 237]	211.55 [130.78; 343.8]	0.704
IL-2	13.53 [11.51; 20.11]	13.41 [11.04; 20.24]	13.65 [11.51; 20.36]	13.89 [12.75; 20.24]	0.752
IL-4	4.49 [2.64; 8.87]	4 [2.45; 8.22]	4.84 [2.75; 10]	6.11 [3.96; 7.39]	0.395
IL-5	81.01 [62.74; 111.02]	77.95 [60.47; 112.95]	85.6 [65.77; 115.67]	87.9 [63.12; 97.89]	0.761
IL-6	7.03 [5.26; 17.07]	6.82 [5.18; 13.69]	12.66 [5.54; 50.59]	10.67 [6.62; 22.7]	0.802
IL-7	29.64 [19.66; 46.49]	31.04 [18.75; 48.67]	26.82 [19.66; 38.31]	35.91 [17.83; 63.79]	0.775
IL-8	21.55 [14.24; 52.92]	18.37 [13.54; 38.81]	53.23 [16.8; 789.8]	47.31 [23.29; 206.99]	0.721
IL-9	289 [200.26; 353.66]	303.15 [196.5; 362.5]	310.2 [200.85; 365.5]	273.2 [201.44; 308.34]	0.421
IL-10	8.33 [7.58; 9.84]	8.33 [7.58; 9.84]	8.7 [7.95; 10.22]	9.27 [8.24; 12.12]	0.417
IL-12	10.75 [10.23; 11.79]	10.75 [9.71; 11.86] ***^c^***	10.75 [10.23; 11.79] ***^c^***	12.05 [11.27; 24.03] ***^a,b^***	**0.035**
IL-13	2.83 [2.4; 3.6]	2.83 [2.4; 3.68]	2.83 [2.4; 3.25]	3.04 [2.34; 3.73]	0.841
IL-15	284.7 [233.1; 321.27]	287.4 [229.4; 327.6]	279.34 [224.2; 311.7]	264.3 [231.61; 288.07]	0.741
IL-17	40.23 [28.61; 62.39]	39.6 [28.21; 65]	41.26 [27.61; 63.92]	40.23 [32.63; 55.24]	0.837
Eotaxin	161.2 [112.1; 226.16]	165 [109.62; 231.84]	153 [116.34; 194.61]	186.6 [108.83; 334.26]	0.637
bFGF	78.74 [67.76; 108.3]	75.91 [59.58; 114.45]	80.97 [69.54; 122.54]	86.44 [71.63; 97.42]	0.357
G-CSF	148 [113.77; 407.9]	136.4 [116.2; 563.6]	191 [118.27; 2077]	230.12 [175; 627.21]	0.475
GM-CSF	6.81 [5.67; 8.7]	6.24 [5.62; 8.91]	7.38 [6; 9.38]	7.71 [6.62; 13.93]	0.468
IFN-γ	18.35 [13.59; 26.77]	18.1 [7.68; 24.76]	18.35 [15.7; 26.9]	20.8 [5.5; 27.27]	0.959
IP-10	1004 [608.7; 1439]	999.5 [526; 1432]	1007 [633.96; 1381]	1027 [595.72; 1897]	0.866
MCP-1	70.44 [50; 100.42]	70.52 [52.59; 105.92]	70.77 [50; 117]	75.26 [55.11; 93.32]	0.926
MIP-1α	4.69 [2.97; 44.63]	3.97 [2.72; 28.32]	6.62 [3.33; 250.59]	8.8 [4.56; 76.46]	0.576
MIP-1β	290.7 [235.75; 397.7]	289.4 [237.5; 360.6]	375.4 [252.2; 825.82]	320.56 [279.84; 407.4]	0.796
PDGF	1985 [1023; 2926]	1952 [878.9; 3004]	1979 [1170; 2711]	2311 [1129; 4226]	0.754
RANTES	20568 [16955; 26172]	21463 [17148; 26258]	20500 [17912; 27167]	18711 [16036; 23079]	0.316
TNF-α	125.49 [99.75; 200.5]	123.65 [96.38; 187]	147.4 [103.56; 259.1]	126.5 [116.56; 167.56]	0.592
VEGF	388.9 [279.3; 506.44]	354.4 [262; 526.14]	390.3 [294.7; 473.12]	396.9 [311.82; 473.12]	0.984
CRP	2.58 [1.14; 4.51]	2.59 [1.17; 4.63]	3.86 [1.44; 10.65]	2.44 [1.22; 5.21]	0.296
Metabolic markers					
C-Peptide	1124 [873.52; 1556]	1155 [798; 1591]	1047 [878.37; 1636]	1252 [1061; 2233]	0.296
Ghrelin	5693 [3745; 9310]	5918 [3815; 9697]	6264 [3917; 9907]	6512 [4249; 13288]	0.748
GIP	310.38 [112.3; 539.3]	324.8 [110.15; 560.3]	308.9 [137.26; 529.5]	479.7 [191.2; 681.6]	0.456
GLP-1	864.5 [256.5; 975.96]	869.4 [249; 969.54]	854.47 [275.8; 935.9]	1040.7 [704.8; 1197.2]	0.107
Glucagon	3098 [1576; 3706]	3007 [1599; 3536]	3098 [1710; 3704]	3633 [1496; 5684]	0.284
Insulin	651.28 [358.28; 1067.98]	659.11 [354.33; 1161]	636.22 [328.16; 1108]	792.38 [516.14; 1089]	0.381
Leptin	12989 [6801; 18758]	17582 [10726; 24410] **^b,c^**	9811 [4574; 15927] ^a^	9163 [4235; 16677] **^a^**	**<0.001**
PAI-1	22173 [13,162; 55273]	20163 [12968; 38209]	25961 [13047; 57125]	27800 [12702; 57104]	0.559
Resistin	6655 [3720; 8725]	6686 [3820; 8473]	6225 [3636; 8767]	6946 [3846; 8473]	0.973
Visfatin	5706 [3450; 8614]	5694 [2743; 8883]	5822 [3901; 8439]	6714 [3789; 10038]	0.758
Adiponectin	4.2 [2.81; 5.78]	4.41 [3.2; 6.44]	3.9 [2.43; 5.77]	4.14 [3.57; 5.09]	0.357

CRP and Adiponectin levels are expressed as mg/l, while every other concentration is reported as pg/ml. All values are reported as median and IQR. p-values in bold are considered statistically significant (p-value <0.05).

a denotes a significant difference versus the BC group; b denotes a significant difference versus the CC group; c denotes a significant difference versus the PC group.

### 3.2 Multifactorial and machine learning-based exploratory analysis

We have investigated the complex association between all patients’ data by using multiple Machine Learning approaches on the overall enrolled population. This strategy was also chosen to avoid potential biases related to small sample subgroup sizes.

Firstly, all parameters evaluated at T0 were stratified in six variable groups (namely “Anagraphic”, “Bioimpedential”, “Clinical”, “Dietary Habits”, “Cytokines”, and “Metabolic Markers”) and subjected to a Multiple Factor Analysis (MFA).

**Figure 1:**
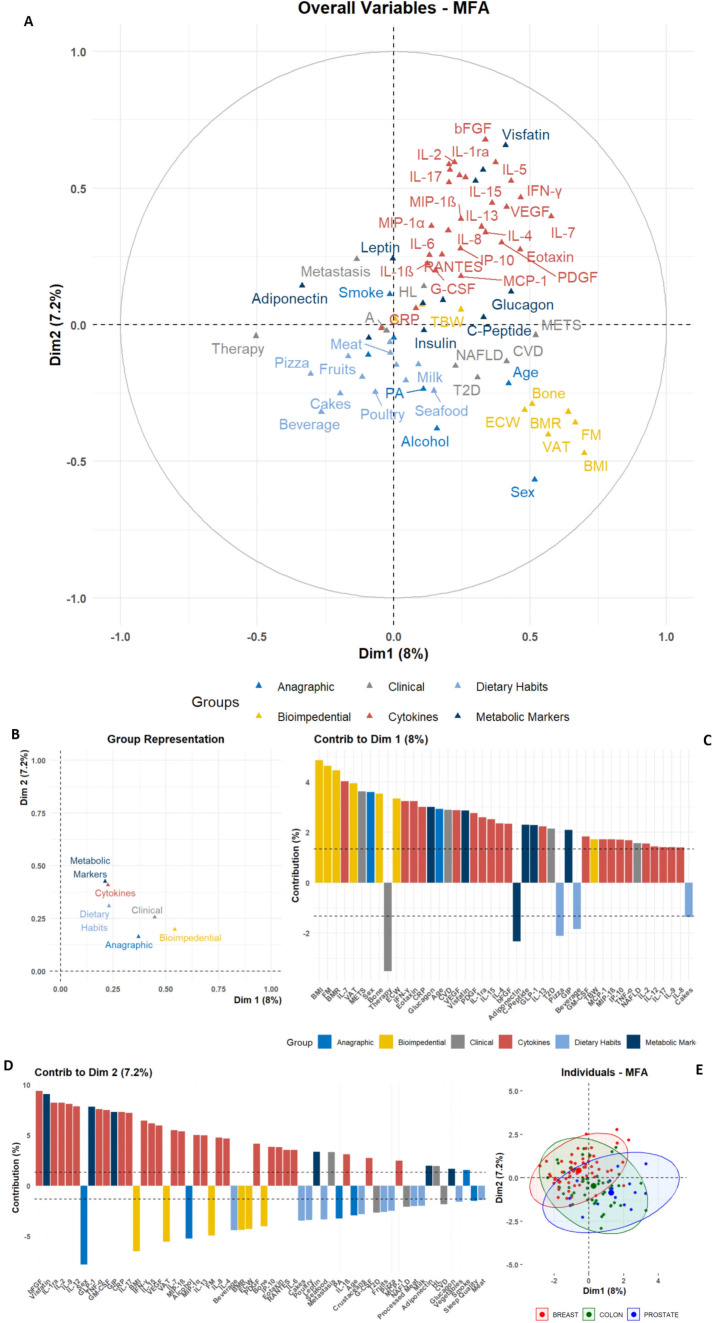
Graphic representation of Multiple Factor Analysis (MFA). The MFA output for anagraphic, biometrical, clinical, dietary, and molecular parameters is reported; **(A)** Biplot depicting the color-coded group variables projections on the two main dimensions (Dim1 and Dim2), allowing the identification of intra- and inter-group clustering. **(B)** Dot plot for groups distributions among Dim1 and Dim2. **(C, D)** Bar plots depicting the single-variable relative contribution to Dim1 and Dim2 variance; the plots only depict variables with an absolute value higher than the mean (dashed line). **(E)** Scatter plot depicting the individual distribution on Dim1 and Dim2, color-coded according to the tumor groups, as shown in the legend.

The overall difference in variables distribution is shown in the biplot, in which very homogeneous clusters of variables can be noted (**Figure 1A**). The variables-group representation also depicts a very strong association among cytokines and metabolic marker distributions, which resulted very close to dietary habits variables; conversely, these molecular markers resulted the furthest from bioimpedential parameters (**Figure 1B**).

The two main dimensions (Dim1 and Dim2) obtained from the MFA allowed to show the contribution of single variables (**Figures 1A, C, D**, **Supplementary Table S3**). In Dim1, the primary contributors were variables from the Bioimpedential group, with BMI, FM, and VAT showing the highest contributions to the overall variance. Additional positive contributors included age and Sex stratification, alongside clinical evidence of METS and CVD. Conversely, variables such as therapy and Adiponectin levels negatively contributed to the association among variables (**Figure 1C**, **Supplementary Table S3**).

In contrast, although cytokines and other molecular markers contributed notably to Dim1, their impact was more pronounced in Dim2, in which bFGF, Visfatin, IL-1ra, IL-2, TNF-α and others had the most substantial positive contribution to variance. Conversely, anagraphic and bioimpedential variables – e.g. Sex, BMI, and VAT – exhibited negative contributions for this dimension’s variance (**Figure 1D**, **Supplementary Table S3**).

This approach revealed latent structures within the multileveled dataset. Notably, Adiponectin was positioned in opposition to a well-grouped cluster of inflammatory cytokines, including Visfatin, IL-6, IL-8, TNF-α, and CRP. At the same time, this inflammatory cluster resulted associated, along Dim1, with bioimpedential markers, such as BMI, FM, and VAT. Consistently, metabolic markers like Insulin and C-Peptide were positively linked to clinical and metabolic variables, such as Glucagon and T2D. Another notable positive association was identified between RANTES, IL-8 and the presence of Metastasis. These associations were further confirmed by the assessment of the single variables to the contribution for MFA dimensions (**Figures 1A, C, D**, **Supplementary Table S3**).

Finally, when the MFA output was clustered for tumor type, no separation among BC, CC, and PC groups was revealed, depicting a heterogeneous global population with no clear neoplastic-specific patterns (**Figure 1E**). The overlapping distribution among the three tumor subgroups was also emphasized after using non-linear dimensionality reduction approaches, such as t-Stochastic Neighbor Embedding (t-SNE) or Uniform Manifold Approximation and Projection (UMAP) (**Supplementary Figure S1**). All techniques, despite the limitations given to the small sample sizes, showed an overall homogeneous dispersion and a lack of proper separation for tumor types.

### 3.3 Dietary intervention evaluation

The population adhered to a Mediterranean-based dietary regimen. After 6 months (T1), bioimpedential data and a sample of serum were collected. At follow-up, a drop-out of 46.97% occurred, with 70 out of 132 starting patients remaining in the study. Baseline comparisons between participants who completed the 6-month intervention and those who discontinued showed no significant differences across demographic, clinical, nutritional, bioimpedance, cytokine, or metabolic parameters, with the only exception for glucagon levels, which resulted higher in patients who completed the intervention compared to dropouts (**Supplementary Table S4**).

**Figure 2:**
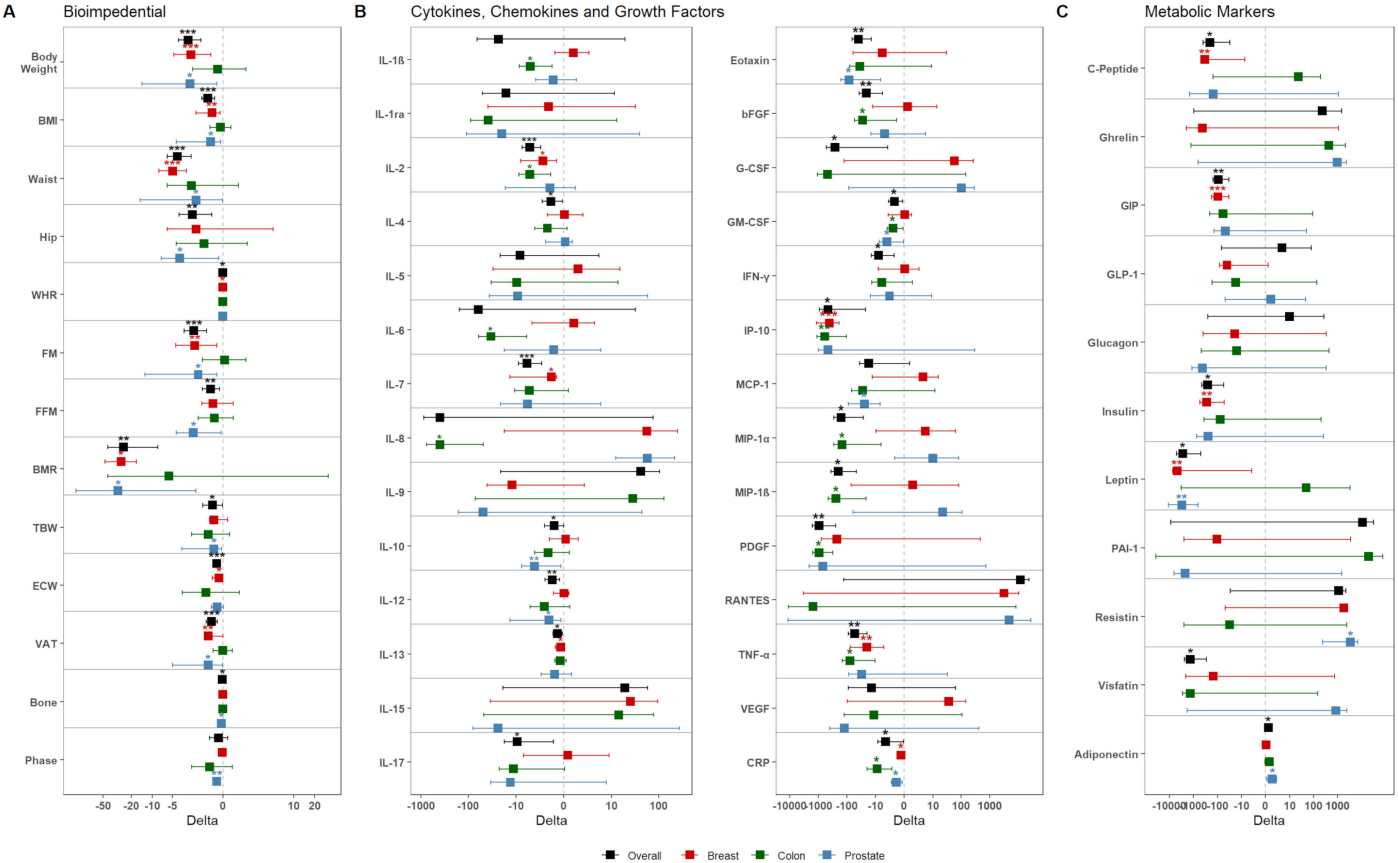
Dietary intervention’s impact on the population. Graphs show the variation of **(A)** bioimpedential, **(B)** cytokines, chemokines, and growth factors, and **(C)** metabolic markers through the overall population and the three tumor subgroups, color-coded accordingly as shown in the legend (black – Overall; red – BC; green – CC; blue – PC). For each parameter, mean variation (square) and 95% confidence intervals (whiskers) between T0 and T1 are represented. Asterisks denote distributions significantly different from zero (*: p-value <0.05; **: p-value <0.01; ***: p-value <0.001).

Overall, upon dietary intervention, patients displayed significant reduction in body weight, BMI, FM, and VAT (all p-values <0.001) (**Figure 2A**, **Supplementary Table S5**). The screening of metabolic markers revealed a significant increase of Adiponectin (p-value: 0.012), accompanied by significant reduction of circulating Insulin (p-value: 0.013), Leptin (p-value: 0.018), and Visfatin (p-value: 0.013) (**Figure 2B**, **Supplementary Table S5**). These outcomes were accompanied by an overall reduction of inflammatory- (such as IL-2, Eotaxin, IFN-γ, IP-10, TNF-α, CRP) and immunomodulatory- (such as IL-4, IL-10, IL-12) molecules, and growth factors (e.g. bFGF, G-CSF, GM-CSF, PDGF) (**Figure 2C**, **Supplementary Table S5**).

Next, we investigated the effects of dietary intervention in the three subgroups. In BC patients, at follow-up, a drop-out of 43.76% was obtained, with 36 out of the 64 patients remaining in the study. Overall, these patients displayed a significant reduction for bioimpedential parameters, such as body weight, BMI, FM, and VAT (**Figure 2A**); these reductions were accompanied with significant reductions for cytokines, including IL-2 (p-value: 0.012), IL-7 (p-value: 0.025), IL-13 (p-value: 0.03), IP-10 (p-value <0.001), TNF-α (p-value: 0.007), and CRP (p-value: 0.038), but also for metabolic markers, such as C-Peptide (p-value: 0.004), GIP (p-value <0.001), Insulin (p-value: 0.005), Leptin (p-value: 0.013) (**Figures 2B, C**, **Supplementary Table S6**).

In regard to the CC subgroup, 25 of the 52 starting patients remained in the study, with a drop-out of 51.93%. Overall, these patients did not display any significant reduction for bioimpedential parameters or metabolic markers; however, they showed significant reduction of inflammatory chemokines and growth factors, among which IL-1β (p-value: 0.018), IL-2 (p-value: 0.025), IL-6 (p-value: 0.02), IL-8 (p-value: 0.027), bFGF (p-value: 0.04), GM-CSF (p-value: 0.023), IP-10 (p-value: 0.005), MIP-1α (p-value: 0.041), MIP-1β (p-value: 0.033), PDGF (p-value: 0.011), TNF-α (p-value: 0.027), and CRP (p-value: 0.02) (**Figures 2B, C**, **Supplementary Table S7**). Notably, all cytokine reductions showed relevant effect size, with Cohen’s d value ranging from -0.60 to -0.97 (**Supplementary Table S7**).

Finally, on the 16 original PC patients, 9 (with a drop-out of 44.75%) remained in the study. Overall, these patients showed significant reduction for various bioimpedential parameters (**Figure 2**); this reduction was accompanied by a significant re-duction of Eotaxin (p-value: 0.027), GM-CSF (p-value: 0.02), MCP-1 (p-value: 0.031), and CRP (p-value: 0.016). Interestingly, these patients also showed reduced levels of Leptin (p-value: 0.08), and increased levels of Adiponectin (p-value: 0.025) and Resistin (p-value: 0.039) (**Figure 2**, **Supplementary Table S8**).

### 3.4 Clinical relevance of nutritionally modified molecules

**Figure 3:**
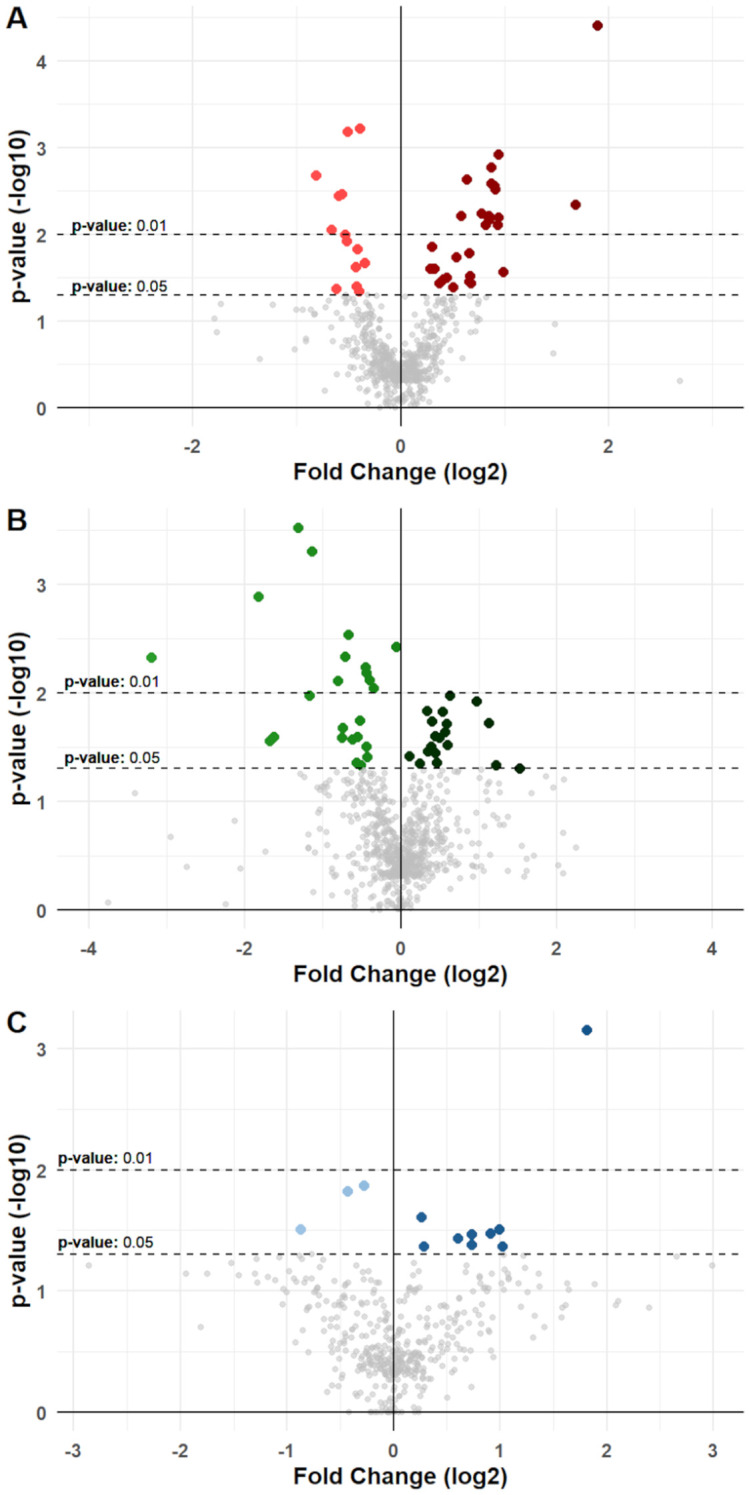
Differential variation for molecules in cancer groups. Volcano plots show differential variations for nutrition-modified cytokines, chemokines, growth factors, and metabolic markers in **(A)** BC, **(B)** CC, and **(C)** PC patients. Each dot represents a protein stratified for a single specific anagraphic, clinical or dietary factor. X-axes represent fold change (log_2_ transformed) and Y-axes represent p-value (-log_10_ transformed). Dotted lines denote thresholds of statistical significance (p-values <0.05 or <0.01). Dots with a brighter color represent a significant reduction for a specific molecule when associated with a specific factor, while dots with a darker color represent a significant increase. Significant comparisons are shown in detail in **Supplementary Tables S6** to **S8** and in **Supplementary File S1**.

We retrospectively investigated the possible associations among these diet-modified molecules with anagraphical, clinical, and nutritional variables at baseline. Univariable comparisons were analyzed between any single molecule with all the aforementioned anagraphic, clinical, and dietary factors, and results were shown in **Figure 3** and **Supplementary File S1**.

In BC patients, univariable analysis displayed a total of 42 significant differences that were noted, with 28 showing an increasing concentration of molecules in association with a specific factor, and 14 showing reductions (**Figure 3**, **Supplementary File S1**). For instance, higher levels of IL-2 in serum of BC patients were associated with presence of T2D (p-value: 0.04); Il-7 and IL-13 were associated with presence of METS (p-value: 0.003 and p-value: 0.005, respectively), CRP was associated with higher levels of BMI and METS (p-value <0.001 and p-value: 0.004, respectively), C-Peptide was associated with higher weekly intake of Milk and dairy products (Mid vs Low intake p-value: 0.087; High vs Low intake p-value: 0.025); GIP and Insulin were likewise associated with higher intake of Milk (p-value: 0.001 and p-value: 0.018, respectively), as well as Cereals (p-value: 0.006 and p-value: 0.016, respectively); finally, Leptin was significantly associated with higher levels of patients’ BMI (p-value: 0.031). These associations were then validated via consecutive univariable and multivariable linear regression models, which confirmed their significance (**Supplementary Table S9**).

In CC patients, 43 significant associations were noted, with 24 reductions and 19 increases (**Figure 3**, **Supplementary File S1**). Notably, a higher intake of fruits resulted associated with reduced levels of inflammatory markers, such as IL-1β (Mid vs Low intake p-value <0.001; High vs Low intake p-value: 0.005), IL-6 (Mid vs Low intake p-value: 0.025; High vs low Intake p-value: 0.011), IL-8 (Mid vs Low intake p-value: 0.027; High vs Low intake p-value: 0.021), MIP-1α (Mid vs Low intake p-value: 0.005; High vs Low intake p-value: 0.001), MIP-1β (Mid vs Low intake p-value: 0.026; High vs Low intake p-value: 0.003), and TNF-α (Mid vs Low intake p-value: 0.018; High vs Low intake p-value <0.001). Other significant associations were noted for BMI with IL-6 (p-value: 0.019) and CRP (p-value: 0.011), for age with IL-1β (p-value: 0.026) and bFGF (p-value: 0.03), and between smoking exposure with IL-8 (p-value: 0.046) (**Figure 3**, **Supplementary File S1**). These associations retained their significance also upon univariable and multivariable linear regression Analysis (**Supplementary Table S10**).

Finally, in PC patients, 12 significant associations were noted, with only 3 reductions and 9 increases (**Figure 3**, **Supplementary File S1**). Eotaxin, CRP, and Leptin levels correlated with BMI (p-values: 0.034, 0.033, and <0.001 respectively); IL-12 (p-value: 0.043) and GM-CSF (p-value: 0.042) were associated with aging; CRP levels were also associated with higher intake of red meats (Low vs Mid intake p-value: 0.031), and finally, physical activity was associated with significantly lower levels of Resistin (p-value: 0.031) (**Figure 3**, **Supplementary Table S11**). These associations were subsequently confirmed through univariable and multivariable linear regression models, maintaining their significance (**Supplementary Table S11**).

## 4 Discussion

Cancer survivors are at high risk of developing metabolic syndrome, obesity, T2D and have an overall reduced quality of life (26–28).

A dietary pattern rich in fruits, vegetables, whole grains, and healthy fats has been shown to modulate inflammatory patterns, reduce oxidative stress, and enhance metabolic homeostasis (29, 30). Furthermore, specific components such as polyphenols, omega-3 fatty acids, and dietary fibers exhibit anti-inflammatory and anti-cancer properties (31, 32). However, how dietary habits may directly impinge on patients with cancer remission has been only partially investigated.

Within this observational study, we have evaluated the impact of a Mediterranean diet-based intervention in patients with different cancers, by analyzing anthropometric data and blood concentration of several cytokines and metabolic hormones. Several studies have aimed to define cancer-specific signatures (33). We have now investigated whether such patterns could be modified by nutritional intervention in patients with cancer remission.

Utilizing machine learning techniques, we demonstrated the presence, at baseline, of a distinct cluster of inflammatory cytokines in the serum of these patients, including Visfatin, IL-6, IL-8, TNF-α, and CRP. A similar cluster, underpinning LGCI, has been previously identified in the serum of blood donors (8). In patients with cancer, the LGCI cytokine cluster was associated with body mass index (BMI), fat mass (FM), and visceral adipose tissue (VAT), and was inversely associated with adiponectin. Additionally, IL-8 and RANTES (also known as CCL5), were found to be associated with the presence of metastasis. This is consistent with other findings, including reports from our research group, describing the involvement of IL-8 and CCL5 in cancer progression and aggressiveness (34, 35). Overall, there were no significant differences in cytokine concentration across tumor subtypes, with the exception of elevated IL-12 levels in prostate cancer (PC). Higher leptin levels, instead, were found in breast cancer (BC). Notably, the nutritional intervention leads to a reduction of both IL-12 and Leptin.

*In vivo* models have demonstrated that IL-12 plays a role in anti-tumor immunity, particularly in prostate cancer (36, 37). Using a multi-modality approach that combines oncolytic viral therapy and IL-12 gene therapy, it has been shown that local administration of IL-12-expressing adenoviruses results in elevated local IL-12 concentrations within the prostate gland, enhancing anti-tumor activity without increasing toxicity. IL-12 stimulates the secretion of multiple cytokines, including IFN-γ, resulting in the activation of innate and adaptive immunity against cancer; however excessive or prolonged IL-12 signaling has been associated with immune-related toxicity and systemic inflammation (38, 39). Indeed, while local IL-12 delivery is under investigation, early clinical trials of systemic IL-12 delivery have been unsuccessful and have resulted in toxicity (36, 37). It is therefore desiderable to induce high levels of IL-12 locally, and reduce the systemic amount of IL-12, as achieved with diet. Leptin has long been recognized as a mediator connecting obesity to BC (40). Leptin levels reflect adipose tissue mass promoting the activation of several oncogenic pathways in BC cells that contribute to cell proliferation, epithelial-mesenchymal transition, stemness, and exosome biogenesis (40, 41). Furthermore, Leptin appears to influence the cancer immune response by inducing a pro-inflammatory immune polarization (40). Our findings confirm that BC is an obesity-associated cancer with a relevant role potentially played by Leptin.

The lack of cancer-specific clustering suggests that metabolism and inflammation exert a broad, common influence on patients, regardless of the primary tumor type. This reinforces the concept that adopting transversal nutritional strategies for managing cancer patients in remission could be beneficial.

In our study, the personalized nutritional intervention led to reductions in BMI, FM, and other anthropometric features where needed (as in BC and PC), while maintaining body weight and bioimpedance data when desired (as in CC). In any case, however, this intervention was associated with a general reduction of the inflammatory milieu.

**Figure 4:**
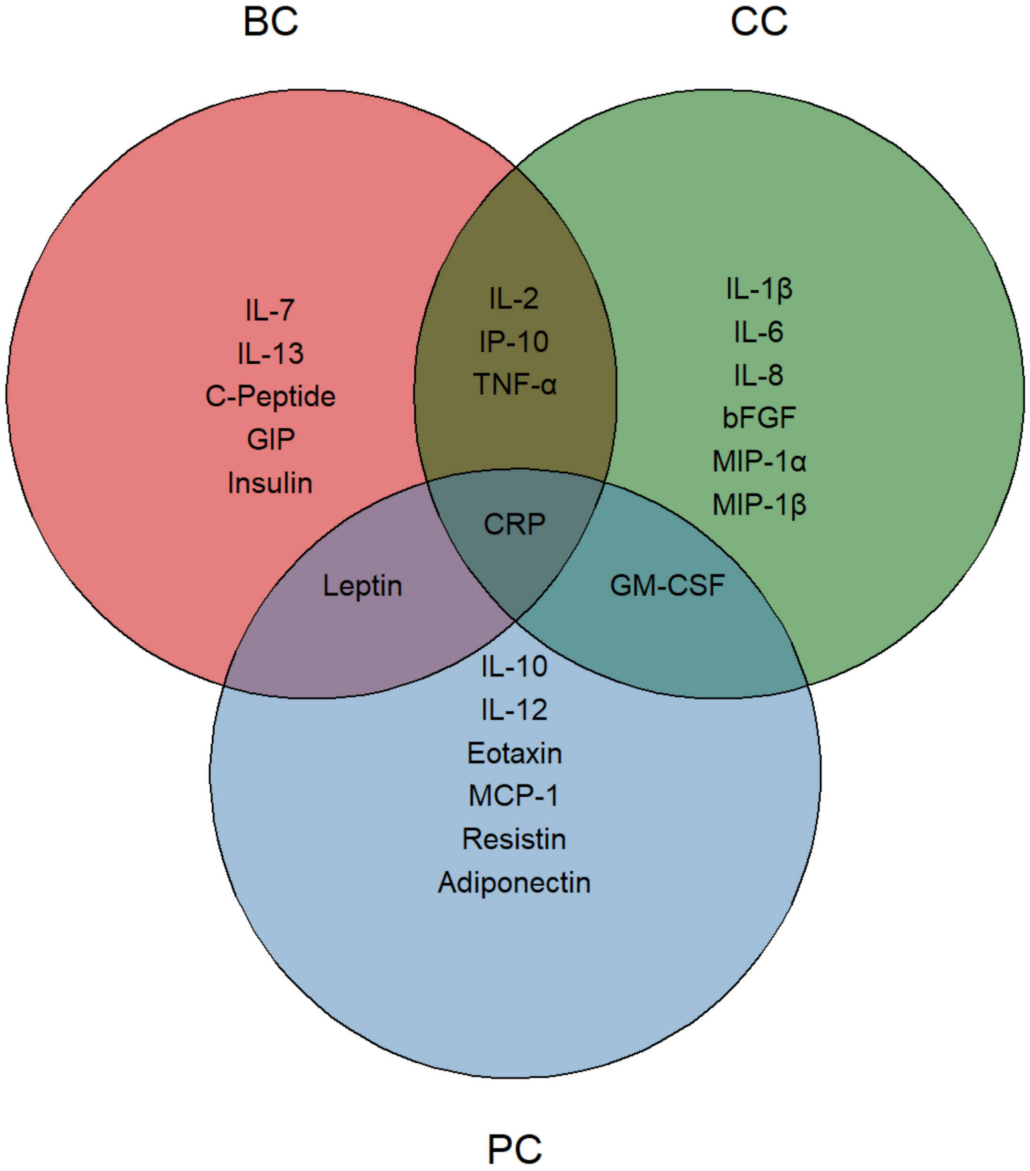
Nutritionally modified cytokines, chemokines, growth factors and metabolic markers. Venn diagram representing the significantly modified circulating molecules. Each circle identifies a specific tumor group, with reduced molecules listed within each area. The intersections highlight significantly modified molecules shared between two or more groups. CRP, positioned at the center, indicates its presence in all three groups.

Overall, CRP was the only common marker that showed a significant reduction across the three subgroups. Leptin, in parallel with patient BMI, was the only marker consistently reduced in BC and PC patients, whereas GM-CSF levels decreased in CC and PC patients. Finally, common reductions of IL-2, IP-10 and TNF-α were observed in BC and CC patients (**Figure 4**). BC and, to a lesser extent, PC patients mainly displayed improvements in metabolic derangements (i.e. C-Peptide, GIP, Insulin), which was consistent with the amelioration of bioimpedential features. At variance, decrease in inflammatory cytokines was marked in CC patients, where changes of anthropometric values were not achieved, neither were they desirable.

To further reinforce this finding, decreased levels of IL-7, IL-13, IP-10, CRP, C-Peptide, Leptin, GIP, and Insulin levels were achieved only in BC patients with weight loss or reduced BMI. However, the only two markers showing a reduction following the dietary intervention, even in the absence of weight loss were TNF-α (p-value: 0.029) and IL-2 (p-value: 0.031). While TNF-α contributes to immune escape and tumor progression by facilitating the biological activity and/or expansion of immunosuppressive cells (42), IL-2 has a dual role since it promotes proliferation and activation of cytotoxic T cells and NK cells, thereby supporting antitumor responses, but may also expand regulatory T cells, potentially dampening immune activation in chronic settings (43, 44). Interestingly, both TNF-α and IL-2 were also reduced in the CC group, suggesting an immunomodulatory effect, independent of weight or fat loss. Notably, most of the markers reduced in BC patients were associated, at baseline, with various metabolic disorders (i.e. METS, T2D, CVD, NAFLD, Hyperlipaemia – **Supplementary Table S9**), highlighting once again the connection between BC and metabolic impairment.

In all cancer groups CRP was associated with patient BMI, in accordance with other studies in healthy and pathological populations (8, 45). Moreover, in PC, higher levels of CRP were associated with higher intake of red meat, as also observed in healthy subjects (8). The nutritional intervention, acting either on patients’ weight or on the controlled consumption of red meat, led to a significant CRP reduction. CRP trajectories have been shown to play an important role in cancer occurrence. A prospective, population-based cohort study with a two-year follow-up revealed that an increasing CRP trajectory pattern was associated with a heightened risk of various cancers, including lung, breast, leukemia, bladder, stomach, colorectal, liver, gallbladder, and extrahepatic bile duct cancer. In contrast, a decreasing CRP trajectory pattern was associated with a reduced risk of esophageal and colorectal cancer (46).

Overall, these findings highlight that a targeted nutritional intervention, characterized by high intake of fruits, vegetables, whole grains, nuts, legumes, olive oil, and seafood, and moderate intake of poultry and dairy, is associated to a reduction of many inflammatory molecules, that are the main players in the connection between inflammation and cancer (47). Different molecular mechanisms may underline the modulation of inflammatory mediators exerted by the diet. For instance, diet significantly influences the composition and diversity of the gut microbiome. A healthy gut microbiome produces beneficial metabolites and modulates immune cell functions, leading to a more balanced cytokine release (48). Approximately 70% of the immune system is located in the gut. Immune cells in the gut are constantly regulated by the microbiome, but also by specific dietary components which directly influence their activity and polarization (e.g., macrophages, T cells, NK cells), thereby impacting cytokine secretion (48, 49). Moreover, anti-inflammatory foods, rich in antioxidants, help neutralize reactive oxygen species (ROS), which are known to contribute to chronic inflammation and cytokine production (48). Finally, diet can influence the metabolic pathways (glycolysis, TCA cycle, OXPHOS) of both cancer cells and immune cells, affecting the availability of substrates for cytokine synthesis and release (49–51). Importantly, dietary interventions can influence not only tissues and cell populations regulating systemic metabolic homeostasis, but also directly modulate tumor-specific immune responses. In fact, metabolites regulate the crosstalk between immune and tumor cells in the tumor microenvironment (TME). TME metabolome is deeply affected by dietary style and TME components (cancer and immune cells) must compete for nutrients to fulfill their metabolic and growth needs, despite tumor origin and stage of disease (52, 53). In conclusion, the molecular mechanisms underlying the effect of dietary intervention on circulating factors are different and complex and still remain to be fully established.

Inflammation is now considered a hallmark of cancer, affecting all stages of cancer, from the initiation of carcinogenesis to metastasis and efficacy of anticancer treatments (47). However, LGCI is also a hallmark of a plethora of non-communicable diseases (i.e. obesity, T2D, METS, NAFLD; hypertension, cardiovascular disease, chronic kidney disease, depression, neurodegenerative and autoimmune diseases, osteoporosis and sarcopenia) (54). Thus, reduced inflammation is beneficial not only for cancer management, but also for ameliorating individuals’ quality of life.

Adherence to the Mediterranean Diet exerts beneficial effects, primarily by reducing inflammatory molecules. Indeed, in all three subgroups of cancer patients, the dietary intervention was accompanied by a significant reduction in CRP levels. In BC and PC patients, who exhibited higher BMI levels at baseline, dietary adherence was associated with a reduction in body weight. In BC patients, inflammatory and immunomodulatory molecules were predominantly linked to metabolic disturbances (e.g., METS, T2D). In prostate cancer, these molecular changes were primarily associated with BMI. Notably, in the colon cancer group, although intervention did not specifically target body weight reduction, a broad spectrum of inflammatory molecules was also reduced, suggesting that the dietary intervention may act through weight-independent immunomodulatory mechanisms. The Mediterranean diet has been shown to modulate the gut–immune axis by shaping the intestinal microbiota and promoting short-chain fatty acid production, which enhance barrier integrity and reduce microbial translocation (48, 49). Such changes can attenuate toll-like receptor signaling on innate immune cells and modulate gut-associated lymphoid tissue (GALT), thereby influencing T-cell polarization and cytokine release (6, 7). Moreover, bioactive components of the diet, such as polyphenols and omega-3 fatty acids, may directly regulate dendritic cell function and antigen presentation, further restraining systemic inflammation (31, 32). These findings reinforce the concept that Mediterranean diet–induced immunoregulation may occur independently of changes in adiposity.

In conclusion, this study offers a multidimensional and in-depth analysis of targeted dietary strategies to potentially optimize the clinical management of cancer patients in remission and reduce the risk of inflammation-related comorbidities.

### 4.1 Limitations

This pilot observational study has different limitations. First, the lack of a control group and randomization, together with the absence of information on potential confounder changes between T0 and T1 (e.g., physical activity, socioeconomic status, medication changes, etc.), restricts the ability to draw causal inferences. Second, the study did not include the assessment of validated Mediterranean diet adherence scores (e.g., PREDIMED, MEDI-LITE), as well as standardized quality-of-life assessment scores (e.g., WHOQOL-BREF, QOLS, EORTC QLQ-C30), which limits comparability with other investigations. Third, methodological constraints related to the intervention should be acknowledged, including a relatively high dropout rate (47% of participants did not complete the 6-month follow-up) and the reliance on nutritionist-administered FFQ at each time point. Finally, although the study investigated circulating inflammatory and metabolic markers, the underlying molecular mechanisms remain unexplored.

## Data Availability

The raw data supporting the conclusions of this article will be made available by the authors, without undue reservation.

## Supplementary material

Supplementary File S1 Nutritionally-impacted molecules and lifestyle factor analysis.
