# Supplementary material for: Unraveling the anti-inflammatory effects of Mediterranean diet in patients with cancer remission
Source: Front Immunol. 2025 Dec 2;16:1666611. doi: 10.3389/fimmu.2025.1666611 (PMC12705368; doi:10.3389/fimmu.2025.1666611)
Supplement: Supplementary File S1 — Nutritionally-impacted molecules and lifestyle factor analysis. [file DataSheet1.pdf]

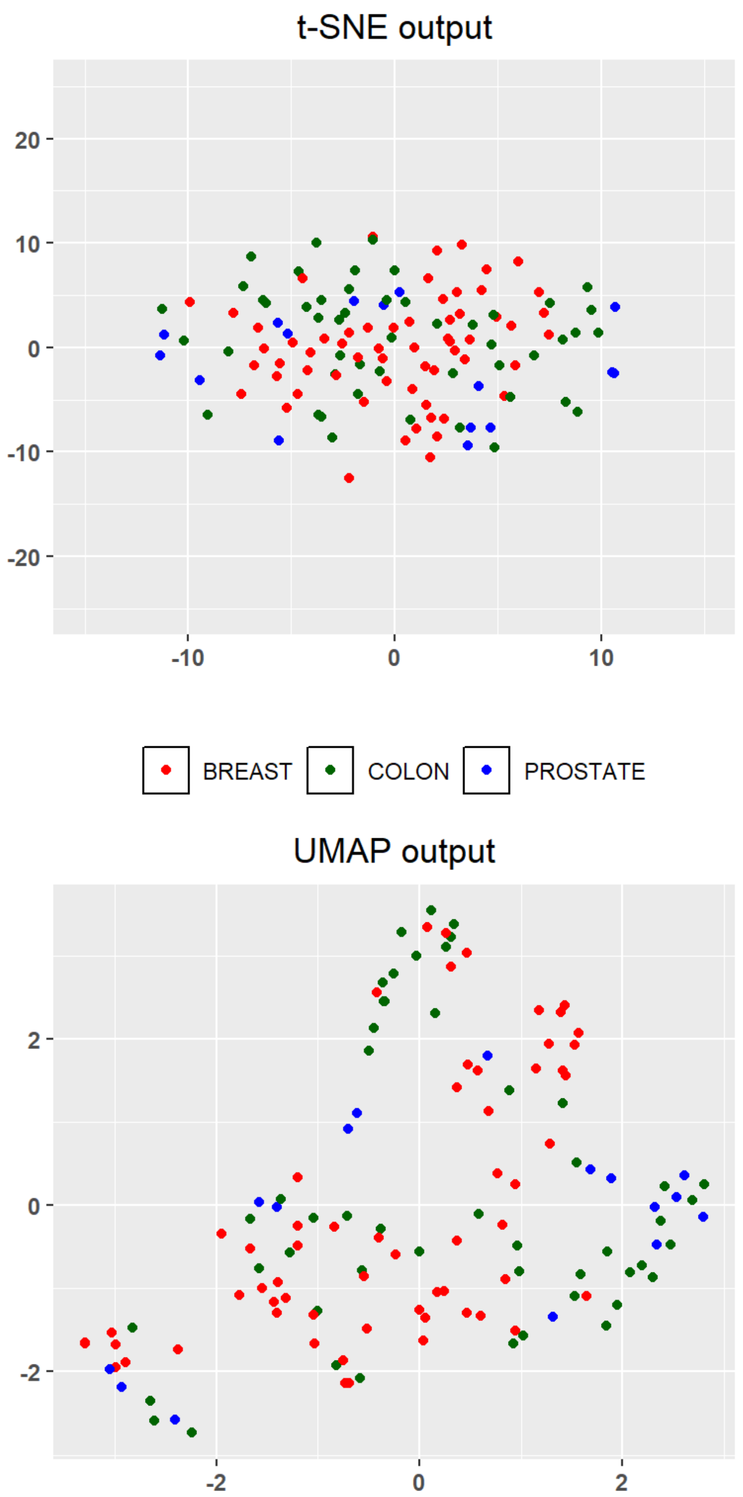

Figure S 1 – t-SNE and UMAP clustering outputs.

Supplementary Table 1: Full Blood Count. Biochemical and hormone analysis of the enrolled population. Data are expressed as median and range [IQR].

Marker	Total (N: 132)	BC (N: 64. 48.48%)	CC (N: 52. 39.39%)	PC (N: 16. 12.12%)
**RBC (10^{12}/L)**	4.58 [4.29; 4.92]	4.40 [4.22; 4.63]	4.77 [4.39; 5.14]	4.84 [4.53; 5.07]
**Hb (g/L)**	13.45 [12.38; 14.33]	13.3 [12.35; 13.8]	14 [12; 15.15]	13.9 [12.6; 14.6]
**Hct (%)**	40.5 [37.75; 43.45]	39.9 [37.55; 41.8]	42.6 [37.92; 45.3]	41.1 [38.92; 44.07]
**WBC (10^9/L)**	6.13 [4.86; 7.06]	6.1 [4.45; 6.93]	6.33 [5.2; 7.63]	6.27 [5.27; 7.06]
**Neutr (10^9/L)**	3.42 [2.60; 4.30]	3.42 [2.57; 4.28]	3.48 [2.64; 4.34]	3.4 [2.72; 4.23]
**Neutr (%)**	58.1 [52.22; 63.80]	60.8 [52.7; 64.75]	57.5 [49.3; 62.75]	56.55 [53.38; 59.53]
**Lympho (10^9/L)**	1.67 [1.36; 2.28]	1.58 [1.21; 2.19]	1.83 [1.4; 2.34]	1.99 [1.43; 2.38]
**Lympho (%)**	29.55 [24.5; 34.7]	27.5 [22.9; 34.85]	30.6 [26.3; 35.25]	31.4 [27.1; 33.92]
**NLR**	1.87 [1.43; 2.54]	2 [1.5; 2.67]	1.85 [1.3; 2.12]	1.74 [1.54; 1.98]
**PLT (10^9/L)**	224 [190; 281]	240 [197; 281]	231 [186.5; 289]	199 [176; 224]
**Insulin (µU/mL)**	9.1 [6.98; 14.81]	9.1 [7.1; 11.95]	12.6 [6.9; 17.12]	11.25 [9.83; 12.67]
**Glycaemia (mg/dL)**	96 [89; 108]	94 [86; 106]	98 [90; 113]	99 [91; 111]
**HOMA IR**	2.47 [1.69; 3.77]	2.06 [1.81; 3.17]	2.16 [1.61; 3.59]	3.6 [3.47; 3.73]
**HbA1c (mmol/mol)**	38 [35; 43]	35 [32.25; 40.81]	38 [35; 43.15]	40.5 [37.5; 44.75]
**HbA1c (%)**	5.6 [5.3; 6.25]	5.75 [5.42; 6.1]	5.55 [5; 5.83]	6.1 [5.6; 6.75]
**Tot Cholesterol (mg/dL)**	196.5 [173.7; 225]	206.5 [183.8; 226.7]	193 [171; 227.5]	179 [162; 196]
**HDL (mg/dL)**	55 [45; 65]	59 [49; 66]	51 [44; 64]	46 [41; 55]
**LDL (mg/dL)**	119.9 [99; 139.68]	122.5 [99; 142]	119.8 [102; 150]	104 [90; 120]
**Triglycerides (mg/dL)**	116 [84; 157.5]	97 [75.75; 145.5]	125 [109; 167.5]	127 [102.5; 154.5]
**ESR (mm/h)**	18 [9; 30]	21 [12; 36.5]	22.5 [9; 29.75]	12 [9; 16]
**AST (U/L)**	20 [18; 25]	20 [18; 24.25]	20.5 [17.25; 26]	20 [17.5; 22.5]
**ALT (U/L)**	21 [15.5; 35]	23 [16; 39]	21 [16; 33.5]	17.5 [15; 20.75]
**CPK (mU/mL)**	68 [52; 106.5]	63.5 [53; 96.75]	84 [50; 108]	68 [57.5; 106.25]
**ALP (U/L)**	77 [67; 98.5]	71 [65; 96.5]	84 [67; 127]	74.5 [69.75; 86.75]
**Total Protein (g/dL)**	7 [6.6; 7.5]	6.9 [6.47; 7.32]	7.3 [6.75; 7.6]	6.7 [6.4; 7.03]
**Albumin (g/dL)**	4 [3.72; 4.32]	4.01 [3.78; 4.32]	3.98 [3.65; 4.32]	3.94 [3.75; 4.31]
**α_2M (g/dL)**	0.76 [0.67; 0.88]	0.75 [0.68; 0.84]	0.76 [0.66; 0.91]	0.76 [0.68; 0.8]
**β_2M (mg/dL)**	0.37 [0.3; 0.47]	0.34 [0.3; 0.4]	0.4 [0.3; 0.62]	0.39 [0.3; 0.47]
**Creatinine (mg/dL)**	0.8 [0.7; 0.92]	0.74 [0.67; 0.82]	0.81 [0.71; 0.9]	1 [0.92; 1.36]
**Azotemia (mg/dL)**	35 [29; 40]	34 [29; 38.75]	35 [28; 41]	48 [37.5; 56.25]
**Cortisol (µg/l)**	11.27 [8.85; 72.35]	11.3 [8.3; 15.3]	10.95 [9.2; 69.72]	75.32 [42.97; 107.66]
**Testosterone (nmol/l)**	2.56 [0.12; 11.32]	-	11.78 [7.17; 16.4]	3.04 [0.16; 10.72]
**E2 (ng/l)**	13.74 [7.22; 19.1]	10 [5; 19.2]	17.9 [17.42; 18.48]	-
**E1 (ng/l)**	9.1 [3.8; 35.33]	6.2 [3; 12]	-	-
**PSA (ng/ml)**	0.66 [0.14; 2.42]	-	1.68 [0.61; 2.87]	0.05 [0.01; 0.48]
**CA 15.3 (U/ml)**	15.2 [11.77; 20.4]	15.6 [11.93; 21.08]	15 [11.4; 16.35]	-
**CA 125 (U/ml)**	9 [5.1; 13.35]	9.2 [6.4; 14.35]	9 [5.05; 11.02]	3.3 [2.95; 3.65]
**CA 19.9 (U/ml)**	9.7 [4; 17]	7.3 [1.99; 13.9]	10.3 [4; 22.32]	13.35 [11.52; 15.18]
**CEA (ng/ml)**	1.6 [1.07; 2.59]	1.2 [0.69; 1.95]	1.86 [1.18; 3.8]	1.73 [1.46; 2.26]
**αFP (ng/ml)**	2.5 [1.6; 3.3]	2.3 [1.5; 2.7]	2.63 [1.7; 3.4]	-
**25(OH)D (ng/ml)**	23.1 [16.91; 31.9]	25 [17.85; 33]	19.9 [16.85; 24.8]	26.55 [17.4; 30.52]

Supplementary Table 2. Dietary habits of the enrolled population (N= 132). Food groups are collected and reported as semi-quantitative variable. Data are reported as count and percentage for the single food groups.

Food Group	Overall (n: 132)	BC (n: 64)	CC (n: 52)	PC (n: 16)
**Cereals**				
Low	39 (29.55%)	24 (37.50%)	13 (25%)	2 (12.5%)
Mid	23 (17.42%)	11 (17.19%)	6 (11.54%)	6 (37.5%)
High	70 (53.03%)	29 (45.31%)	33 (63.46%)	8 (50%)
**Milk**				
Low	43 (32.58%)	25 (39.06%)	16 (30.77%)	2 (12.5%)
Mid	35 (26.52%)	18 (28.12%)	10 (19.23%)	7 (43.75%)
High	54 (40.91%)	21 (32.81%)	26 (50%)	7 (43.75%)
**Seafood**				
Low	10 (7.58%)	6 (9.38%)	3 (5.77%)	1 (6.25%)
Mid	94 (71.21%)	47 (73.44%)	34 (65.38%)	13 (81.25%)
High	28 (21.21%)	11 (17.19%)	15 (28.85%)	2 (12.5%)
**Crustaceans**				
Low	111 (84.09%)	57 (89.06%)	40 (76.92%)	14 (87.5%)
High	21 (15.91%)	7 (10.94%)	12 (23.08%)	2 (12.5%)
**Meat**				
Low	26 (19.70%)	12 (18.75%)	10 (19.23%)	4 (25%)
Mid	92 (69.70%)	46 (71.88%)	34 (65.38%)	12 (75%)
High	14 (10.61%)	6 (9.38%)	8 (15.38%)	0 (0%)
**Poultry**				
Low	14 (10.61%)	7 (10.94%)	5 (9.62%)	2 (12.5%)
Mid	94 (71.21%)	47 (73.44%)	36 (69.23%)	11 (68.75%)
High	24 (18.18%)	10 (15.62%)	11 (21.15%)	3 (18.75%)
**Processed Meat**				
Low	38 (28.79%)	20 (31.25%)	13 (25%)	5 (31.25%)
Mid	76 (57.58%)	38 (59.38%)	32 (61.54%)	6 (37.5%)
High	18 (13.64%)	6 (9.38%)	7 (13.46%)	5 (31.25%)
**Vegetables**				
Low	24 (18.18%)	10 (15.62%)	11 (21.15%)	3 (18.75%)
Mid	38 (28.79%)	18 (28.12%)	16 (30.77%)	4 (25%)
High	70 (53.03%)	36 (56.25%)	25 (48.08%)	9 (56.25%)
**Fruits**				
Low	28 (21.21%)	17 (26.56%)	9 (17.31%)	2 (12.5%)
Mid	31 (23.48%)	13 (20.31%)	12 (23.08%)	6 (37.5%)
High	73 (55.30%)	34 (53.12%)	31 (59.62%)	8 (50%)
**Cakes**				
Low	16 (12.12%)	7 (10.94%)	8 (15.38%)	1 (6.25%)
Mid	26 (19.70%)	18 (28.12%)	4 (7.69%)	4 (25%)
High	90 (68.18%)	39 (60.94%)	40 (76.92%)	11 (68.75%)
**Pizza**				
Low	49 (37.12%)	22 (34.38%)	19 (36.54%)	8 (50%)
Mid	19 (14.39%)	11 (17.19%)	6 (11.54%)	2 (12.5%)
High	64 (48.48%)	31 (48.44%)	27 (51.92%)	6 (37.5%)
**Beverage**				
Low	38 (28.79%)	17 (26.56%)	15 (28.85%)	6 (37.5%)
Mid	15 (11.36%)	8 (12.5%)	7 (13.46%)	0 (0%)
High	79 (59.85%)	39 (60.94%)	30 (57.69%)	10 (62.5%)

Supplementary Table 3 – MFA output and relative contributions of single variables to Dim1 and Dim2.

Variable	Group	Contribution to Dim1 (%)	Contribution to Dim2 (%)
**Sex**	Anagraphic	3.6001	-7.8299
**Age**	Anagraphic	2.9306	-2.9617
**Sleep Time**	Anagraphic	0.0014	-0.6713
**Physical Activity**	Anagraphic	0.7554	-3.2523
**Smoke**	Anagraphic	-0.0935	1.5397
**Alcohol**	Anagraphic	1.1074	-5.2606
**Sleep Quality**	Anagraphic	-0.6450	-1.5207
**Metastasis**	Clinical	-0.9393	3.3273
**Therapy**	Clinical	-3.5124	-0.5717
**METS**	Clinical	3.6213	-0.5212
**T2D**	Clinical	2.1453	-2.6678
**CVD**	Clinical	2.8856	-1.8520
**NAFLD**	Clinical	1.5713	-2.0882
**Hyperlipaemia**	Clinical	0.7758	1.9428
**Anemia**	Clinical	-0.1818	-0.3061
**BMI**	Bioimpedential	4.8606	-6.4980
**FM**	Bioimpedential	4.6390	-4.9550
**FFM**	Bioimpedential	0.7258	1.0153
**BMR**	Bioimpedential	4.4545	-4.4004
**TBW**	Bioimpedential	1.7212	0.7715
**ECW**	Bioimpedential	3.3424	-4.3067
**VAT**	Bioimpedential	3.9454	-5.5462
**Bone**	Bioimpedential	3.5381	-4.0154
**Phase**	Bioimpedential	0.0383	0.2761
**PDGF**	Cytokines	2.7584	4.1501
**IL-1ß**	Cytokines	0.8564	3.0935
**IL-1ra**	Cytokines	2.5961	8.2182
**IL-2**	Cytokines	1.5472	8.2159
**IL-4**	Cytokines	2.3472	4.6769
**IL-5**	Cytokines	0.5691	0.8408
**IL-6**	Cytokines	0.9081	3.5213
**IL-7**	Cytokines	4.0206	5.4825
**IL-8**	Cytokines	1.3919	4.7675
**IL-9**	Cytokines	1.4058	8.1062
**IL-10**	Cytokines	-0.3151	-0.1647
**IL-12**	Cytokines	1.4401	7.8459
**IL-13**	Cytokines	2.2377	4.9571
**IL-15**	Cytokines	2.5147	6.1619
**IL-17**	Cytokines	1.4147	7.2037
**Eotaxin**	Cytokines	3.2337	3.8044
**bFGF**	Cytokines	2.3388	9.3636
**G-CSF**	Cytokines	1.0707	2.7500
**GM-CSF**	Cytokines	1.8296	7.4657
**IFN-γ**	Cytokines	3.2399	6.4410
**IP-10**	Cytokines	1.7094	3.8542

**MCP-1**	Cytokines	1.7186	2.4537
**MIP-1α**	Cytokines	0.9580	4.9929
**MIP-1ß**	Cytokines	1.7157	5.3562
**RANTES**	Cytokines	1.2287	3.5443
**TNF-α**	Cytokines	1.6799	7.5614
**VEGF**	Cytokines	2.8834	5.9491
**CRP**	Cytokines	3.0015	7.2825
**Cereals**	Dietary Habits	-0.1035	-0.8865
**Milk**	Dietary Habits	0.6320	-2.0029
**Seafood**	Dietary Habits	1.0232	-3.3360
**Crustaceans**	Dietary Habits	0.3134	-2.8153
**Meat**	Dietary Habits	-0.0904	-1.4279
**Poultry**	Dietary Habits	-0.4672	-3.3971
**Processed Meat**	Dietary Habits	0.0645	-2.0405
**Vegetables**	Dietary Habits	-1.1598	-1.6038
**Fruits**	Dietary Habits	-0.7975	-2.6288
**Cakes**	Dietary Habits	-1.3662	-3.4774
**Pizza**	Dietary Habits	-2.1201	-2.4813
**Beverage**	Dietary Habits	-1.8454	-4.4081
**C-Peptide**	Metabolic Markers	2.2998	0.3770
**Ghrelin**	Metabolic Markers	-0.6315	-0.6593
**GIP**	Metabolic Markers	2.0935	7.2874
**GLP-1**	Metabolic Markers	2.2811	7.8256
**Glucagon**	Metabolic Markers	3.0007	1.6657
**Insulin**	Metabolic Markers	0.7746	-0.2826
**Leptin**	Metabolic Markers	-0.0194	3.3532
**PAI-1**	Metabolic Markers	0.7492	1.0956
**Resistin**	Metabolic Markers	1.2576	1.2529
**Visfatin**	Metabolic Markers	2.8611	9.0713
**Adiponectin**	Metabolic Markers	-2.3381	1.9751

Supplementary Table 4. Baseline Comparison of completer and dropout patients.			
**Marker**	**Complete (n: 70)**	**Dropouts (n: 62)**	**p-value**
**Anagraphic Features**			
**Sex (Females. %)**	45 (34.35%)	39 (29.77%)	0.999
**Age (Years)**	59.27 ± 10.94	62.93 ± 11.04	0.121
<50 (%)	13 (18.57%)	11 (17.75%)	
50-59 (%)	27 (38.57%)	12 (19.35%)	0.025
60-69 (%)	17 (24.29%)	22 (35.48%)	
≥70 (%)	13 (18.57%)	17 (27.42%)	
**Height (m)**	1.65 [1.57; 1.71]	1.63 [1.58; 1.67]	0.464
**Weight (Kg)**	77.6 [67.75; 88.32]	78.2 [66.4; 86.3]	0.901
**Phys. Act. (%)**	32 (45.71%)	23 (37.10%)	0.377
**Smokers (%)**	18 (25.72%)	11 (18.03%)	0.399
**Alcohol (%)**	24 (34.28%)	19 (31.15%)	0.714
**Sleep Time (hrs)**	6.21 ± 1.51	6.28 ± 1.43	0.803
**Sleep Quality (%)**			
Low (%)	32 (45.72%)	24 (38.71%)	0.571
Medium (%)	19 (27.14%)	16 (25.81%)	
High (%)	19 (27.14%)	22 (35.48%)	
**Bioimpedential Features**			
**BMI (Kg/m^2)**	28.55 [24.97; 32.51]	28.56 [25.45; 32.81]	0.804
NW (%)	17 (25.72%)	15 (24.19%)	
OW (%)	26 (37.14%)	22 (35.48%)	0.926
OB (%)	26 (37.14%)	26 (40.33%)	
**Waist (cm)**	100.75 [93.25; 111.5]	103.25 [95.38; 113.62]	0.366
**Hip (cm)**	105.5 [100.47; 114.75]	106.75 [102; 120.25]	0.130
**WHR**	0.95 [0.90; 1.01]	0.95 [0.88; 1]	0.482
**FM (Kg)**	25.1 [19.45; 32.25]	24.1 [17.3; 33.05]	0.796
**FFM (Kg)**	49.8 [46.2; 57.05]	51.7 [46.3; 55.5]	0.481
**BMR (kcal)**	1498 [1361; 1654]	1542 [1402; 1656]	0.605
**TBW (L)**	35 [31.55; 39.15]	34.8 [31.95; 40.25]	0.577
**ECW (L)**	16.7 [14.7; 18.2]	16.8 [15.15; 18.35]	0.504
**VAT (Kg)**	10 [7; 12]	10 [7; 12]	0.783
**Bone (Kg)**	2.6 [2.3; 2.85]	2.6 [2.3; 2.8]	0.302
**Phase (°)**	5.4 [5.1; 5.95]	5.2 [4.9; 5.9]	0.103
**Clinical Features**			
**Metastasis (%)**	7 (10%)	12 (19.35%)	0.143
**Chemoterapy (%)**	26 (37.14%)	21 (33.87%)	0.718
**METS (%)**	8 (11.43)	6 (9.68%)	0.785
**T2D (%)**	7 (10%)	14 (22.58%)	0.059
**CVD (%)**	35 (50%)	30 (48.39%)	0.863
**NAFLD (%)**	18 (25.72%)	15 (24.19%)	0.999
**Hyperlipaemia (%)**	35 (50%)	24 (38.71%)	0.222
**Anemia (%)**	5 (7.14%)	2 (3.23%)	0.447
**Dietary Habits**			
**Cereals**			
Low	16 (22.86%)	23 (37.1%)	0.109
Mid	11 (15.72%)	12 (19.35%)	
High	43 (61.43%)	27 (43.54%)	
**Milk**			0.074
Low	16 (22.86%)	27 (43.55%)	

Mid	19 (27.14%)	16 (25.81%)	
High	35 (50%)	19 (30.64%)	
**Seafood**			
Low	9 (12.86%)	1 (1.61%)	0.043
Mid	50 (71.43%)	44 (70.97%)	
High	11 (15.71%)	17 (27.42%)	
**Crustaceans**			
Low	57 (81.43%)	54 (87.1%)	0.476
High	13 (18.57%)	8 (12.9%)	
**Meat**			
Low	14 (20%)	12 (19.35%)	0.347
Mid	46 (65.71%)	46 (74.19%)	
High	10 (14.29%)	4 (6.46%)	
**Poultry**			
Low	9 (12.86%)	5 (8.06%)	0.671
Mid	48 (68.57%)	46 (74.19%)	
High	13 (18.57%)	11 (17.74%)	
**Processed Meat**			
Low	21 (30%)	17 (27.42%)	0.939
Mid	40 (57.14%)	36 (58.06%)	
High	9 (12.86%)	9 (14.52%)	
**Vegetables**			
Low	14 (20%)	10 (16.13%)	0.842
Mid	19 (27.14%)	19 (30.65%)	
High	37 (52.86%)	33 (53.22%)	
**Fruits**			
Low	14 (20%)	14 (22.58%)	0.488
Mid	14 (20%)	17 (27.42%)	
High	42 (60%)	31 (50%)	
**Cakes**			
Low	11 (15.71%)	5 (8.06%)	0.443
Mid	13 (18.57%)	13 (20.97%)	
High	46 (65.72%)	44 (70.97%)	
**Pizza**			
Low	22 (31.43%)	27 (43.55%)	0.298
Mid	10 (14.29%)	9 (14.52%)	
High	38 (54.28%)	26 (41.93%)	
**Beverage**			
Low	26 (37.14%)	12 (19.35%)	0.077
Mid	7 (10%)	8 (12.9%)	
High	37 (52.86%)	42 (67.75%)	
**Cytokines. Chemokines. and Growth Factors**			
**IL-1β**	4.76 [3.96; 10.28]	5.58 [3.79; 12.76]	0.367
**IL-1ra**	208.28 [164.03; 338.98]	171.09 [128.87; 310.87]	0.323
**IL-2**	13.17 [11.51; 21.18]	14.61 [11.51; 20.93]	0.318
**IL-4**	4.24 [2.83; 7.34]	6.13 [2.59; 9.74]	0.171
**IL-5**	81.01 [62.74; 109.86]	84.07 [62.74; 123.82]	0.361
**IL-6**	7.24 [5.38; 30.59]	12.82 [6.1; 45.59]	0.529
**IL-7**	35.21 [21.11; 47.50]	21.11 [18.20; 43.76]	0.059
**IL-8**	36.64 [16.37; 227.36]	79.97 [16.51; 873.60]	0.298
**IL-9**	273.92 [195.09; 349.53]	311.64 [235.19; 360.16]	0.148
**IL-10**	9.08 [7.95; 11.38]	8.89 [7.95; 10.51]	0.318
**IL-12**	11.40 [10.23; 12.84]	10.75 [10.23; 12.25]	0.303

**IL-13**	2.83 [2.61; 4.05]	2.61 [2.18; 3.65]	0.232
**IL-15**	272.56 [220.52; 317.44]	284.72 [229.4; 322.54]	0.780
**IL-17**	35.48 [27.01; 67.42]	48.41 [34.47; 63.04]	0.266
**Eotaxin**	177.22 [130.96; 230.54]	152.73 [90.34; 209.09]	0.166
**bFGF**	78.74 [69.54; 106.87]	79.85 [62.21; 128.84]	0.396
**G-CSF**	168.13 [118.82; 691.24]	333.16 [121.22; 1584]	0.083
**GM-CSF**	7.57 [5.86; 11.05]	7.38 [5.67; 10.27]	0.663
**IFN-γ**	19.08 [15.94; 28.06]	17.68 [9.75; 28.06]	0.212
**IP-10**	1138.37 [750.84; 1870]	849.93 [509.37; 1405]	0.061
**MCP-1**	66.18 [46.94; 101.3]	77.82 [55.84; 118.74]	0.773
**MIP-1α**	4.88 [3.22; 78.53]	23.52 [3.18; 270.97]	0.657
**MIP-1β**	288.86 [239.22; 404.80]	402.87 [275.19; 782.68]	0.832
**PDGF**	2498 [1333; 3856]	1591 [898; 2628]	0.089
**RANTES**	22119 [18424; 27049]	21236 [16193; 26538]	0.263
**TNF-α**	130.72 [102.64; 211.65]	158.06 [103.25; 244.99]	0.102
**VEGF**	367.42 [295.46; 507.11]	401.60 [271.47; 516.99]	0.949
**CRP**	3.73 [1.71; 6.35]	2.69 [1.36; 6.92]	0.606
**Metabolic Markers**			
**C-Peptide**	1485 [951.33; 2232]	1318 [843; 2420]	0.601
**Ghrelin**	6553 [4794; 10897]	4958 [2303; 9310]	0.571
**GIP**	477.72 [297.37; 630.30]	207.68 [91.78; 358.05]	0.261
**GLP-1**	909.72 [834.61; 1175]	787.72 [202.36; 886.58]	0.354
**Glucagon**	3335 [2804; 5335]	2715 [1468; 3481]	0.006
**Insulin**	1016 [607.36; 1661]	539.29 [314.08; 880.01]	0.155
**Leptin**	15138 [6934; 22209]	12989 [5626; 20836]	0.694
**PAI-1**	24707 [18438; 54618]	16608 [11854; 56716]	0.117
**Resistin**	6382 [3924; 8250]	6698 [3363; 10852]	0.289
**Visfatin**	7127 [4970; 11378]	3959 [2863; 6812]	0.412
**Adiponectin**	3.93 [2.76; 5.62]	4.58 [3.08; 6.34]	0.807

Supplementary Table 5 – Impact of Dietary Intervention on overall population. All distributions are referred to the 70 patients. p-values are referred to paired comparisons among T0 and T1. Effect size is described with Cohen's d and its 95% CI. Emboldened p-values are considered statistically significant (<0.05).

Parameters	T0	T1	Δ (95% CI)	p-val	Cohen's d (95% CI)
**Body Weight (Kg)**	77.6 [68.35; 87.92]	75.7 [66.6; 83.1]	-2.82 (-4.07 – -1.57)	**<0.001**	-0.23 (-0.33 – -0.12**)**
**BMI (Kg/m^2)**	28.65 [25.32; 32.67]	27.83 [24.24; 31.4]	-1.02 (-1.48 – -0.55)	**<0.001**	-0.21 (-031 – -0.11)
**Waist (cm)**	102.5 [93.75; 111.5]	97 [90; 105]	-4.22 (-5.96 – -2.49)	**<0.001**	-0.36 (-0.51 – -0.21)
**Hip (cm)**	106 [100.83; 115.25]	106 [98.5; 112]	-2.36 (-3.98 – -0.74)	**0.005**	-0.24 (-0.40 – -0.08)
**WHR**	0.95 [0.89; 1.01]	0.91 [0.87; 0.96]	-0.02 (-0.03 – -0.01)	**0.012**	-0.25 (-0.44 – -0.06)
**FM (Kg)**	25.5 [19.55; 33.25]	23.8 [16.88; 30.05]	-2.19 (-3.28 – -1.11)	**<0.001**	-0.24 (-0.36 – -0.12)
**FFM (Kg)**	49.6 [46.05; 56.8]	50.05 [45.1; 54.9]	-0.84 (-1.46 – -0.22)	**0.008**	-0.11 (-0.19 – -0.03)
**BMR (kcal)**	1476 [1359; 1642]	1465 [1343; 1623]	-25.63 (-43.06 – -8.2)	**0.005**	-0.12 (-0.19 – -0.04)
**TBW (L)**	34.5 [31.35; 38.2]	34.45 [31.6; 38.8]	-0.71 (-1.39 – -0.03)	**0.041**	-0.12 (-0.24 – -0.01)
**ECW (L)**	16.7 [14.7; 18.15]	16.2 [14.7; 17.83]	-0.4 (-0.65 – -0.15)	**<0.001**	-0.22 (-0.44 – -0.01)
**VAT (Kg)**	10 [7; 12]	9 [7; 11]	-0.76 (-1.15 – -0.37)	**<0.001**	-0.22 (-0.33 – -0.10)
**Bone (Kg)**	2.5 [2.3; 2.8]	2.5 [2.3; 2.8]	-0.04 (-0.07 – -0.01)	**0.018**	-0.09 (-0.18 – -0.02)
**Phase (°)**	5.4 [5.1; 5.85]	5.4 [5; 6.1]	-0.3 (-0.92 – 0.32)	0.333	-0.15 (-0.45 – 0.15)
**IL-2 (pg/ml)**	13.17 [11.51; 20.24]	11.98 [11.04; 13.77]	-4.99 (-7.27 – -2.7)	**<0.001**	-0.74 (-1.11 – -0.36)
**IL-4 (pg/ml)**	4.24 [2.79; 7.49]	3.83 [3.08; 6.08]	-1.31 (-2.49 – -0.13)	**0.03**	-0.37 (-0.71 – -0.03)
**IL-7 (pg/ml)**	34.52 [21.11; 47.5]	32.03 [21.47; 36.6]	-5.69 (-8.80 – -2.57)	**<0.001**	-0.36 (-0.56 – -0.16)
**IL-10 (pg/ml)**	9.08 [7.81; 11.38]	8.7 [7.95; 9.84]	-0.97 (-2.16 - -0.01)	**0.035**	-0.25 (-0.58 – -0.08)
**IL-12 (pg/ml)**	11.4 [10.23; 12.84]	10.75 [9.71; 11.27]	-1.17 (-2.08 - -0.39)	**0.003**	-0.29 (-0.63 – -0.05)
**IL-13 (pg/ml)**	2.83 [2.61; 3.84]	2.61 [2.4; 2.83]	-0.61 (-1.09 – -0.12)	**0.015**	-0.39 (-0.71 – -0.07)
**IL-17 (pg/ml)**	35.48 [27.01; 69.32]	34.06 [27.51; 43.35]	-9.32 (-17.59 – -1.05)	**0.028**	-0.38 (-0.74 – -0.03)
**Eotaxin (pg/ml)**	177.22 [132.61; 227.88]	138.98 [93.18; 195.48]	-40 (-65.78 – -14.22)	**0.002**	-0.39 (-0.65 – -0.13)
**bFGF (pg/ml)**	78.74 [69.54; 109.93]	74.19 [67.46; 81.79]	-21.42 (-37.35 – -5.49)	**0.009**	-0.45 (-0.80 – -0.10)
**G-CSF (pg/ml)**	168.13 [117.04; 973.53]	238.24 [120.31; 734.56]	-266.26 (-529 – -3.52)	**0.047**	-0.31 (-0.62 – -0.01)
**GM-CSF (pg/ml)**	7.57 [5.86; 10.11]	6.43 [5.86; 8.3]	-1.76 (-3.23 – -0.29)	**0.019**	-0.43 (-0.81 – -0.06)
**IFN-γ (pg/ml)**	19.08 [15.94; 28.33]	19.08 [15.94; 22.77]	-7.9 (-13.97 – -1.82)	**0.011**	-0.38 (-0.68 – -0.08)
**IP-10 (pg/ml)**	1138 [763.17; 1791]	767.22 [553.59; 1070]	-475.3 (-928.6 – 22.04)	**0.04**	-0.30 (-0.60 – -0.01)
**MIP-1α (pg/ml)**	4.88 [3.15; 161.56]	19.73 [3.49; 105.71]	-161 (-294.6 – -27.26)	**0.019**	-0.37 (-0.69 – -0.06)
**MIP-1β (pg/ml)**	288.86 [235.04; 426.03]	302.65 [254.22; 397.28]	-204.1 (-359.9 – -48.26)	**0.011**	-0.39 (-0.69 – -0.08)
**PDGF (pg/ml)**	2498 [1405; 3764]	1528 [939.9; 3233]	-962 (-1678 – -245.9)	**0.009**	-0.38 (-0.66 – -0.09)
**TNF-α (pg/ml)**	130.72 [101.12; 225.5]	111.67 [89.88; 145.49]	-55.2 (-90.44 – -19.92)	**0.003**	-0.54 (-0.91 – -0.17)
**CRP (mg/dl)**	4.06 [1.74; 6.38]	3.21 [1.28; 5.39]	-4.23 (-8.32 – -0.14)	**0.043**	-0.25 (-0.49 – -0.01)
**C-Peptide (pg/ml)**	1485 [934.66; 2187]	1212 [757.51; 1761]	-210.8 (-390.8 – 30.8)	**0.022**	-0.21 (-0.39 – -0.03)
**GIP (pg/ml)**	477.7 [301.92; 641.8]	419.8 [242.; 508.95]	-91.9 (-150.96 – -32.8)	**0.003**	-0.21 (-0.35 – -0.08)
**Insulin (pg/ml)**	1016 [612.39; 1601]	823.52 [559.49; 1197]	-254.65 (-455.4 – -53.9)	**0.013**	-0.28 (-0.51 – -0.06)
**Leptin (pg/ml)**	15761 [6987; 22297]	12119 [5631; 21428]	-2803 (-5104 – -501.71)	**0.018**	-0.19 (-0.34 – -0.03)
**Visfatin (pg/ml)**	7127 [5451; 10812]	7194 [4081; 8873]	-1345 (-2396 – 294.29)	**0.013**	-0.24 (-0.43 – -0.05)
**Adiponectin (mg/dl)**	3.93 [2.8; 5.76]	4.14 [3.38; 6.51]	0.56 (0.12 – 1.01)	**0.012**	0.17 (0.04 – 0.29)

Supplementary Table 6 – Impact of Dietary Intervention on BC population. All distributions are referred to the 36 patients. p-values are referred to paired comparisons among T0 and T1. Effect size is described with Cohen's d and its 95% CI. Emboldened p-values are considered statistically significant (<0.05).

Parameters	T0	T1	Δ (95% CI)	p-val	Cohen's d (95% CI)
**Body Weight (Kg)**	72.8 [66.6; 88.6]	73.6 [64.9; 84.5]	-2.5 (-4.8 – -0.8)	**<0.001**	-0.22 (-0.45 – -0.01)
**BMI (Kg/m^2)**	29 [26.2; 33.7]	28.1 [24.4; 32.8]	-0.734 (-2.0 – -0.190)	**0.003**	-0.19 (-0.38 – -0.01)
**Waist (cm)**	99.3 [89.9; 114]	94 [85.8; 107]	-5.0 (-8 – -3.0)	**<0.001**	-0.41 (-0.61 – -0.21)
**Hip (cm)**	107 [102; 118]	108 [98.9; 118]	-2.0 (-6.0 – 5.0)	0.084	-0.21 (-0.44 – 0.03)
**WHR**	0.905 [0.883; 0.958]	0.89 [0.863; 0.925]	-0.018 (-0.03 – 0.0)	**0.019**	-0.44 (-0.86 – -0.01)
**FM (Kg)**	25.7 [21.1; 35.2]	25.1 [20; 31.5]	-2.1 (-4.5 – -0.4)	**0.002**	-0.27 (-0.47 – -0.07)
**FFM (Kg)**	47.9 [44.7; 52.5]	48.1 [44; 51.6]	-0.65 (-1.5 – 0.7)	0.207	-0.03 (-0.30 – 0.24)
**BMR (kcal)**	1452 [1335; 1591]	1455 [1328; 1539]	-28 (-47 – 17)	**0.017**	-0.17 (-0.36 – -0.09)
**TBW (L)**	32 [30.1; 35.3]	32 [30.2; 35]	-0.6 (-0.9 – 0.3)	0.120	-0.21 (-0.34 – 0.29)
**ECW (L)**	15.7 [14.3; 17.5]	15.5 [14; 17]	-0.25 (-0.7 - -0.1)	**0.034**	-0.13 (-0.42 – -0.04)
**VAT (Kg)**	8 [7; 11]	7.5 [5.75; 9.25]	-1 (-1 – 0)	**0.001**	-0.27 (-0.44 – -0.1)
**Bone (Kg)**	2.45 [2.28; 2.7]	2.4 [2.2; 2.6]	0 (-0.1 – 0)	0.057	-0.06 (-0.34 – 0.21)
**Phase (°)**	5.4 [5.18; 5.9]	5.5 [5.08; 6.1]	-0.05 (-0.2 – 0.1)	0.764	0.02 (-0.44 – 0.48)
**IL-2 (pg/ml)**	12.7 [11; 21.2]	12.1 [11.5; 13.4]	-2.34 (-7.95 – -0.71)	**0.012**	-0.66 (-1.15 – -0.16)
**IL-7 (pg/ml)**	36.3 [23.1; 52.9]	33.1 [21.5; 38.1]	-1.25 (-13.3 - -0.69)	**0.025**	-0.44 (-0.75 – -0.14)
**IL-13 (pg/ml)**	2.99 [2.61; 4.26]	2.83 [2.4; 3.04]	-0.27 (-0.85 – -0.01)	**0.03**	-0.27 (-0.70 – -0.01)
**IP-10 (pg/ml)**	1193 [803; 2515]	815 [530; 1070]	-423 (-1189 – -194)	**<0.001**	-0.84 (-1.31 – -0.36)
**TNF-α (pg/ml)**	143 [110; 194]	114 [89.8; 145]	-20.6 (-77.5 – -4.91)	**0.007**	-0.72 (-1.27 – -0.16)
**CRP (mg/dl)**	3.47 [1.12; 6.16]	2.3 [1.18; 4.69]	-0.49 (-1.14 – -0.04)	**0.038**	-0.19 (-0.51 – -0.04)
**C-Peptide (pg/ml)**	1506 [963; 2297]	1369 [763; 1732]	-342 (-497 – -7.17)	**0.004**	-0.26 (-0.77 – -0.06)
**GIP (pg/ml)**	490 [284; 660]	424 [243; 570]	-96.6 (-175 – -33)	**<0.001**	-0.38 (-0.61 – -0.16)
**Insulin (pg/ml)**	1169 [670; 2048]	867 [602; 1259]	-282 (-546 - -52.7)	**0.005**	-0.41 (-0.80 – -0.02)
**Leptin (pg/ml)**	18136 [13611; 30284]	14866 [6057; 25468]	-4815 (-7425 – 3.37)	**0.013**	-0.30 (-0.53 – -0.07)

Supplementary Table 7 – Impact of Dietary Intervention on CC population. All distributions are referred to the 25 patients. p-values are referred to paired comparisons among T0 and T1. Effect size is described with Cohen's d and its 95% CI. Emboldened p-values are considered statistically significant (<0.05).

Parameters	T0	T1	Δ (95% CI)	p-val	Cohen's d (95% CI)
**Body Weight (Kg)**	75 [62.2; 83.6]	73 [61.8; 81.4]	-0.337 (-2.32 – 1.65)	0.394	-0.03 (-0.18 – 0.13)
**BMI (Kg/m^2)**	26 [23.9; 29.8]	25.9 [23.5; 30.2]	-0.168 (-0.88 – 0.54)	0.439	-0.04 (-0.20 – 0.12)
**Waist (cm)**	100 [89.9; 107]	97 [87.8; 103]	-2.47 (-5.99 – 1.05)	0.086	-0.20 (-0.49 – 0.08)
**Hip (cm)**	101 [97; 106]	102 [94.3; 107]	-1.3 (-4.37 – 1.77)	0.267	-0.16 (-0.52 – 0.20)
**WHR**	0.99 [0.91; 1.03]	0.96 [0.89; 1.04]	-0.01 (-0.03 – 0.008)	0.199	-0.23 (-0.50 – 0.05)
**FM (Kg)**	19.4 [10.6; 26.9]	19.6 [14.9; 27.2]	0.11 (-1.45 – 1.67)	0.84	0.01 (-0.16 – 0.18)
**FFM (Kg)**	52.3 [44.6; 62]	53.8 [45.1; 59.6]	-0.57 (-1.82 – 0.68)	0.341	-0.07 (-0.21 – 0.08)
**BMR (kcal)**	1515 [1329; 1805]	1507 [1341; 1771]	-5.7 (-42.8 – 31.4)	0.818	-0.02 (-0.17 – 0.12)
**TBW (L)**	37 [32.8; 44.5]	36.3 [32; 42.1]	-0.99 (-2.42 – 0.425)	0.19	-0.14 (-0.34 – 0.06)
**ECW (L)**	16.7 [15.1; 19.5]	16.5 [15; 19.1]	-1.17 (-3.49 – 1.14)	0.473	-0.26 (-0.77 – 0.25)
**VAT (Kg)**	10 [7; 13]	10 [7; 13]	0 (-0.639 – 0.639)	0.784	0 (-0.17 – 0.17)
**Bone (Kg)**	2.6 [2.3; 3.1]	2.7 [2.3; 3]	-0.01 (-0.079 – 0.054)	0.779	-0.03 (-0.20 – 0.13)
**Phase (°)**	5.3 [5.1; 6.4]	5.4 [5; 6.3]	-0.891 (-2.41 – 0.629)	0.615	-0.29 (-0.78 – 0.20)
**IL-1β (pg/ml)**	5.65 [4.18; 13.5]	4.07 [2.63; 6.01]	-4.79 (-8.4 – -1.17)	**0.018**	-0.97 (-1.83 – -0.10)
**IL-2 (pg/ml)**	13.4 [11.3; 22]	12 [10.8; 13.8]	-4.95 (-8.57 – -1.33)	**0.025**	-0.90 (-1.65 – -0.14)
**IL-6 (pg/ml)**	13.1 [6; 88.7]	7.65 [4.77; 19.4]	-33.9 (-61.9 – -5.85)	**0.02**	-0.79 (-1.52 – -0.06)
**IL-8 (pg/ml)**	37.6 [15.8; 751]	51.4 [14.7; 118]	-399 (-749 – -49.2)	**0.027**	-0.79 (-1.56 – -0.02)
**bFGF (pg/ml)**	81 [69.5; 116]	70.7 [60.7; 81.5]	-28.3 (-55.2 – -1.34)	**0.04**	-0.76 (-1.57 – -0.04)
**GM-CSF (pg/ml)**	7.76 [6.05; 9.64]	6.05 [5.48; 7.67]	-2.09 (-3.62 – -0.19)	**0.023**	-0.57 (-1.20 – -0.05)
**IP-10 (pg/ml)**	1049 [629; 1365]	684 [467; 968]	-617 (-1129 – -104)	**0.005**	-0.64 (-1.22 – -0.07)
**MIP-1α (pg/ml)**	4.78 [3.37; 267]	10.3 [3.31; 44.4]	-149 (-292 – -6.33)	**0.041**	-0.64 (-1.31– -0.02)
**MIP-1β (pg/ml)**	312 [237; 968]	302 [247; 380]	-243 (-465 – -21.7)	**0.033**	-0.60 (-1.17 – -0.02)
**PDGF (pg/ml)**	2255 [1229; 3758]	1133 [779; 2233]	-961 (-1612 – -309)	**0.011**	-0.79 (-1.39 – -0.19)
**TNF-α (pg/ml)**	146 [100; 274]	104 [76; 144]	-78.6 (-147 – -10.4)	**0.027**	-0.81 (-1.60 – -0.02)
**CRP (mg/dl)**	4.34 [2.75; 14]	3.71 [2.76; 7.94]	-8.92 (-20.2 – 2.36)	**0.02**	-0.29 (-0.65 – -0.07)

Supplementary Table 8 – Impact of Dietary Intervention on PC population. All distributions are referred to the 9 patients. p-values are referred to paired comparisons among T0 and T1. Effect size is described with Cohen's d and its 95% CI. Emboldened p-values are considered statistically significant (<0.05).

Parameters	T0	T1	Δ (95% CI)	p-val	Cohen's d (95% CI)
**Body Weight (Kg)**	86.1 [77; 94.1]	79.1 [70.2; 87.8]	-2.6 (-14 – -0.4)	**0.031**	-0.54 (-1.08 – 0.01)
**BMI (Kg/m²)**	29.7 [25.5; 30.7]	26 [23.5; 29.5]	-0.82 (-4.41 – -0.137)	**0.039**	-0.47 (-0.96 – -0.02)
**Waist (cm)**	110 [100; 115]	108 [92; 114]	-2 (-15 – -0.01)	**0.031**	-0.41 (-0.82 – -0.01)
**Hip (cm)**	106 [103; 107]	100 [97; 108]	-3.86 (-7.45 – -0.27)	**0.039**	-0.67 (-1.29 – -0.06)
**WHR**	1.03 [0.98; 1.08]	1.05 [0.96; 1.07]	0.005 (-0.009 – 0.05)	0.938	-0.06 (-0.93 – 0.81)
**FM (Kg)**	22.1 [19.9; 28.3]	20.2 [16.9; 24.6]	-1.8 (-12.8 – -0.4)	**0.037**	-0.72 (-1.57 – -0.13)
**FFM (Kg)**	63.8 [56.1; 66.1]	62.2 [52.1; 64]	-2.24 (-4.39 – -0.103)	**0.039**	-0.33 (-0.63 – -0.03)
**BMR (kcal)**	1829 [1546; 1924]	1781 [1514; 1850]	-31 (-122 – -2)	**0.027**	-0.28 (-0.57 – -0.01)
**TBW (L)**	44.2 [40.1; 46.7]	41.1 [38.4; 45]	-0.6 (-3.6 – -0.1)	**0.031**	-0.43 (-0.85 – -0.13)
**ECW (L)**	19.5 [17.4; 20.5]	19.1 [17.3; 20.3]	-0.367 (-0.773 – 0.04)	0.086	-0.21 (-0.43 – 0.01)
**VAT (Kg)**	14 [11.5; 15.5]	12 [9.5; 14.5]	-1 (-5 – -0.02)	**0.039**	-0.59 (-1.25 – -0.07)
**Bone (Kg)**	3.2 [2.7; 3.3]	3.1 [2.65; 3.15]	-0.1 (-0.177 – -0.023)	**0.031**	-0.27 (-0.46 – -0.08)
**Phase (°)**	5.5 [5.15; 5.7]	5.2 [4.6; 5.35]	-0.4 (-0.658 – -0.142)	**0.008**	-0.66 (-1.09 – -0.23)
**IL-10 (pg/ml)**	9.84 [8.52; 19]	7.95 [7.31; 10.4]	-3.87 (-7.46 – -0.27)	**0.008**	-0.63 (-1.21 – -0.04)
**IL-12 (pg/ml)**	12.3 [11.3; 24.8]	10.8 [9.97; 12.1]	-1.56 (-13.3 – -0.26)	**0.02**	-0.62 (-1.22 – -0.02)
**Eotaxin (pg/ml)**	199 [150; 364]	179 [95.7; 245]	-85.9 (-165 – -6.67)	**0.027**	-0.49 (-0.94 – -0.05)
**GM-CSF (pg/ml)**	8.51 [7.33; 15.3]	6.05 [5.86; 8.37]	-3.71 (-7.35 – -0.07)	**0.02**	-0.94 (-1.96 – -0.08)
**MCP-1 (pg/ml)**	76.4 [42.4; 108]	31.4 [26.5; 53.6]	-24.9 (-92 – -6.88)	**0.031**	-0.71 (-1.42 – -0.01)
**CRP (mg/dl)**	3.13 [1.32; 5.41]	1.39 [0.972; 3]	-1.39 (-2.48 – -0.297)	**0.016**	-0.65 (-1.15 – -0.14)
**Leptin (pg/ml)**	13595 [5027; 21275]	6557 [1922; 18689]	-3097 (-11155 – -635)	**0.008**	-0.35 (-0.79 – -0.10)
**Resistin (pg/ml)**	7194 [4019; 8647]	8764 [5721; 14809]	3451 (232 – 6670)	**0.039**	0.86 (0.01 – 1.72)
**Adiponectin (mg/dl)**	3.56 [2.29; 3.85]	5.01 [3.23; 6.09]	1.37 (0.263 – 2.48)	**0.025**	0.27 (0.13 – 0.68)

Supplementary Table 9 – Univariate and Multivariate model analysis for BC patients at T0. Table reports association estimates and their 95% CI between cytokines, chemokines, growth factors, metabolic markers and anagraphical, clinical and nutritional variables. For each association, both univariate and multivariate data are reported. Only data for molecules with a significant reduction between T0 and T1 are shown. Emboldened p-values are considered statistically significant (<0.05).

Marker	Univariate Model			Multivariate Model		
		Estimate (95% CI)	P-val		Estimate (95% CI)	P-val
**IL-2**	**METS**	16.05 (1.10 – 19.03)	**0.036**	**METS**	1.46 (0.22 – 11.79)	0.662
	**T2D**	6.55 (0.95 – 12.14)	**0.023**	**T2D**	6.00 (5.18 – 8.1)	**0.042**
	**CVD**	3.39 (0.01 – 6.77)	**0.049**	**CVD**	3.04 (-0.45 – 6.52)	0.086
**IL-7**	**Metastasis**	-15.38 (-29.89 – -0.91)	**0.037**	**Metastasis**	-12.66 (-23.91 – -1.41)	**0.028**
	**Therapy**	-12.36 (-21.04 – -3.68)	**0.006**	**Therapy**	-12.0 (-19.12 – -4.88)	**0.001**
	**METS**	28.53 (12.94 – 44.13)	**<0.001**	**METS**	15.74 (2.38 – 29.1)	**0.022**
	**Cakes**			**Cakes**		
	Mid	-4.28 (-19.55 – 10.98)	0.577	Mid	-7.04 (-19.68 – 5.6)	0.269
	High	-17.42 (-31.49 – 3.35)	**0.017**	High	-13.43 (-25.19 – -1.67)	**0.026**
	**Beverages**			**Beverages**		
	Mid	8.0 (-7.69 – -0.66)	0.264	Mid	2.83 (-9.33 – 15)	0.643
	High	-13.84 (-10.15 – -2.12)	**0.005**	High	-10.17 (-18.75 – -1.59)	**0.021**
**IL-13**	**Metastasis**	-1.08 (-2.04 – -0.11)	**0.029**	**Metastasis**	-0.94 (-1.78 – -0.11)	**0.028**
	**Therapy**	-0.82 (-1.38 – -0.26)	**0.005**	**Therapy**	-0.60 (-1.11 – -0.09)	**0.021**
	**METS**	1.41 (0.38 – 2.44)	**0.008**	**METS**	0.94 (0.01 – 1.87)	**0.047**
	**Cakes**			**Cakes**		
	Mid	-0.99 (-1.98 – -0.01)	**0.049**	Mid	-1.01 (-1.88 – -0.13)	**0.026**
	High	-1.39 (-2.28 – -0.49)	**0.003**	High	-1.26 (-2.06 – -0.46)	**0.002**
**IP-10**	**Age**	410.2 (14.41 – 806)	**0.042**	**Age**	10.56 (-7.68 – 28.79)	0.251
	**Therapy**	-340.8 (-741.38 – 59.76)	**0.031**	**Therapy**	-167.54 (-561.14 – 226.06)	0.397
	**Cereals**			**Cereals**		
	Mid	685.3 (163.97 – 1206.67)	**0.011**	Mid	598.75 (64.22 – 1133.29)	**0.029**
	High	623.1 (203.7 – 1042.45)	**0.004**	High	543.1 (105.31 – 980.89)	**0.016**
**TNF-α**	**METS**	107.88 (37.88 – 177.89)	**0.003**	**METS**	70.53 (-8.63 – 149.7)	0.080
	**T2D**	85.25 (13.04 – 157.46)	**0.021**	**T2D**	62.42 (-12.04 – 136.88)	0.099
	**CVD**	9.08 (1.58 – 80.33)	**0.048**	**CVD**	26.36 (-15.62 – 68.34)	0.213
**CRP**	**BMI**	0.34 (0.24 – 0.45)	**<0.001**	**BMI**	0.24 (0.14 – 0.35)	**<0.001**
	**METS**	6.38 (4.03 – 8.74)	**<0.001**	**METS**	3.19 (0.87 – 5.50)	**0.008**
	**NAFLD**	2.40 (1.06 – 3.74)	**<0.001**	**NAFLD**	1.05 (-0.03 – 2.13)	0.057
**C-Peptide**	**Therapy**	-317.2 (-631.68 – 2.68)	**0.048**	**Therapy**	-373.4 (-675.61 – -71.23)	**0.016**
	**Milk**			**Milk**		
	Mid	273.3 (-95.08 – 641.61)	0.143	Mid	298.7 (-54.63 – 651.93)	0.095
	High	453.9 (66.75 – 841.04)	**0.022**	High	516.1 (142.04 – 890.22)	**0.008**
**GIP**	**Cereals**			**Cereals**		
	Mid	144.47 (-42.63 – 331.57)	0.128	Mid	129.82 (-51.31 – 310.96)	0.157
	High	177.32 (33.69 – 320.95)	**0.016**	High	142.18 (1.05 – 283.31)	**0.048**
	**Milk**			**Milk**		
	Mid	140.44 (-17.48 – 298.36)	0.08	Mid	120.43 (-35.55 – 276.41)	0.127
	High	219.46 (68.01 – 370.92)	**0.005**	High	191.7 (40.29 – 343.11)	**0.014**
**Insulin**	**Cereals**			**Cereals**		
	Mid	472.9 (91.46 – 854.39)	**0.016**	Mid	404.5 (35.2 – 773.9)	**0.032**
	High	341.1 (29.62 – 652.55)	**0.032**	High	303.1 (-0.95 – 607.09)	0.051
	**Milk**			**Milk**		
	Mid	487.1 (141.19 – 833.07)	**0.007**	Mid	431.8 (95.55 – 767.96)	**0.013**
	High	272.4 (-53.65 – 598.54)	0.099	High	209.1 (-110.61 – 528.71)	0.195
**Leptin**	**BMI**	494.8 (49.1 – 940.59)	**0.03**	**BMI**	431.1 (295.1 – 865.15)	**0.008**
	**Hyperlipaemia**	-5218 (-9837 – -599.24)	**0.028**	**Hyperlipaemia**	-3839 (-8327 – -649.7)	**0.023**
	**Pizza**			**Pizza**		
	Mid	-2587 (-9501 – 4237)	0.456	Mid	-2190 (-8677 – 4297)	0.209
	High	-6674 (-11941 – -1407)	**0.014**	High	-6803 (-11750 – -1857)	**<0.001**

Supplementary Table 10 – Univariate and Multivariate model analysis for CC patients at T0. Table reports association estimates and their 95% CI between cytokines, chemokines, growth factors, metabolic markers and anagraphical, clinical and nutritional variables. For each association, both univariate and multivariate data are reported. Only data for molecules with a significant reduction between T0 and T1 are shown. Emboldened p-values are considered statistically significant (<0.05).

Marker	Univariate Model			Multivariate Model		
		Estimate (95% CI)	P-val		Estimate (95% CI)	P-val
**IL-1β**	**Age**	0.08 (0.01 – 0.23)	**0.039**	**Age**	0.11 (0.02 – 0.26)	**0.013**
	**Fruits**			**Fruits**		
	Mid	-6.31 (-9.74 – -2.87)	**<0.001**	Mid	-6.69 (-10.08 - -3.30)	**0.001**
	High	-4.12 (-7.19 – -1.04)	**0.009**	High	-4.63 (-7.69 - -1.56)	**0.029**
**IL-6**	**BMI**	3.95 (0.36 – 7.55)	**0.031**	**BMI**	4.11 (0.49 – 7.73)	**0.026**
	**Crustaceans**	13.07 (3.50 – 22.66)	**<0.001**	**Crustaceans**	8.09 (-2.27 – 18.46)	0.121
	**Fruits**			**Fruits**		
	Mid	-19.26 (-31.68 - -6.85)	**0.003**	Mid	-15.12 (-28.73 – -1.51)	**0.03**
	High	-15.82 (-27.25 - -4.38)	**0.008**	High	-12.27 (-24.57 – -0.02)	**0.05**
**IL-8**	**Smoke**	115.18 (16.02 – 246.38)	**0.038**	**Smoke**	133.1 (1.53 – 264.66)	**0.047**
	**Fruits**			**Fruits**		
	Mid	-84.29 (-347.03 - -60.83)	**0.034**	Mid	-120.59 (-307.97 - -66.78)	**0.021**
	High	-143.1 (-276.29 – 107.70)	**0.002**	High	-176.38 (-374.47 – 21.71)	**0.001**
**bFGF**	**Age**	1.40 (0.06 – 2.73)	**0.041**	**Age**	1.56 (0.30 – 2.82)	**0.016**
	**CVD**	-28.39 (-50.42 - -6.35)	**0.013**	**CVD**	-24.31 (-46.36 – -2.26)	**0.031**
	**Fruits**			**Fruits**		
	Mid	-3.99 (-35.23 – 0.90)	0.091	Mid	-8.17 (-62.57 – -7.1)	**0.036**
	High	-34.8 (-70.50 – -27.24)	**0.004**	High	-27.73 (-37.86 – -21.52)	**0.005**
**GM-CSF**	**Fruits**			**Fruits**		
	Mid	-2.54 (-4.22 – -0.87)	**0.004**	Mid	-2.27 (-3.93 - -0.62)	**0.008**
	High	-2.30 (-3.79 – -0.81)	**0.003**	High	-1.75 (-3.32 - -0.19)	**0.029**
	**Cakes**			**Cakes**		
	Mid	-1.76 (-4.22 - -0.87)	0.119	Mid	-1.48 (-3.59 – 0.64)	0.165
	High	-1.92 (-3.79 - -0.01)	**0.049**	High	-1.50 (-2.96 – 0.35)	0.143
**IP-10**	**Age**	19.35 (2.97 – 35.72)	**0.022**	**Age**	13.62 (-3.14 – 30.38)	0.095
	**Meat**			**Meat**		
	Mid	-203 (-566.52 – 160.46)	0.267	Mid	-118.89 (-482.26 – 244.47)	0.407
	High	-546.8 (-1017 - -77.16)	**0.023**	High	-329.44 (-808.16 – 149.27)	0.088
	**Cakes**			**Cakes**		
	Mid	-71.85 (-687.99 – 544.29)	0.728	Mid	-182.23 (-771.30 – 406.85)	0.456
	High	-352.69 (-756.2 – 50.83)	**0.032**	High	-305.89 (-693.01 – 81.24)	0.065
	**Pizza**			**Pizza**		
	Mid	-45.13 (-531.91 – 441.64)	0.853	Mid	-51.26 (-534.99 – 432.46)	0.865
	High	-325.85 (-618.15 – -33.55)	**0.030**	High	-224.14 (-513.85 – 65.56)	0.119
**MIP-1α**	**Smoke**	48.72 (23.99 – 121.42)	**0.021**	**Smoke**	62.11 (0.21 – 123.99)	**0.049**
	**Crustaceans**	77.36 (10.13 – 144.59)	**0.025**	**Crustaceans**	26.86 (-39.58 – 93.29)	0.418
	**Fruits**			**Fruits**		
	Mid	-144.70 (-221.94 – -67.45)	**<0.001**	Mid	-137.91 (-221.24 – -54.58)	**0.002**
	High	-116.61 (-186.48 – -46.74)	**<0.001**	High	-111.06 (-185.06 – -37.05)	**0.004**
**MIP-1β**	**Fruits**			**Fruits**		
	Mid	-223.72 (-419.63 - -27.81)	**0.026**	Mid	-	-
	High	-204.60 (-382.41 - -26.78)	**0.025**	High	-	-
**TNF-α**	**Crustaceans**	59.31 (0.55 – 118.06)	**0.048**	**Crustaceans**	16.23 (-51.08 – 83.54)	0.629
	**Processed Meat**			**Processed Meat**		
	Mid	39.97 (-13.96 – 110.46)	0.376	Mid	20.81 (-39.85 – 81.47)	0.493
	High	48.25 (23.46 – 126.17)	**0.046**	High	2.61 (-84.86 – 90.08)	0.953
	**Fruits**			**Fruits**		
	Mid	-76.29 (-141.62 – -10.96)	**0.023**	Mid	-64.27 (-142.29 – 13.75)	0.081
	High	-136.48 (-210.86 – -62.10)	**<0.001**	High	-120.13 (-209.43 – -30.83)	**0.009**
**CRP**	**BMI**	0.27 (0.13 – 1.68)	**0.007**	**BMI**	-	-

Supplementary Table 11 – Univariate and Multivariate model analysis for PC patients at T0. Table reports association estimates and their 95% CI between cytokines, chemokines, growth factors, metabolic markers and anagraphical, clinical and nutritional variables. For each association, both univariate and multivariate data are reported. Only data for molecules with a significant reduction between T0 and T1 are shown. Emboldened p-values are considered statistically significant (<0.05).

Marker	Univariate Model			Multivariate Model		
		Estimate (95% CI)	P-val		Estimate (95% CI)	P-val
**IL-10**	**NAFLD**	1.64 (0.69 – 2.58)	**0.003**	**NAFLD**	1.88 (0.49 – 3-28)	**0.014**
	**Cereals**			**Cereals**		
	Mid	-1.23 (-2.75 – 0.29)	0.100	Mid	0.66 (-1.12 – 2.44)	0.418
	High	-1.76 (-3.20 – -0.33)	**0.021**	High	-0.19 (-1.75 – 1.37)	0.786
**IL-12**	**Age**	0.37 (0.09 – 0.65)	**0.012**	**Age**	-	-
**Eotaxin**	**BMI**	13.37 (0.87 – 25.87)	**0.038**	**BMI**	-	-
**GM-CSF**	**Age**	0.19 (0.02 – 0.35)	**0.029**	**Age**	0.18 (0.05 – 0.31)	**0.013**
	**Beverage**			**Beverage**		
	Mid	-	-	Mid	-	-
	High	-2.28 (-4.69 - -0.14)	**0.038**	High	-2.14 (-3.94 – -0.33)	**0.025**
**CRP**	**BMI**	0.73 (0.02 – 4.39)	**0.047**	**BMI**	0.81 (0.15 – 1.07)	**0.041**
	**Meats**			**Meats**		
	Mid	5.05 (0.73 – 9.38)	**0.025**	Mid	2.58 (0.81 – 4.03)	**0.014**
	High	-	-	High	-	-
**Leptin**	**BMI**	1216 (624.85 – 1808)	**<0.001**	**BMI**	-	-
**Resistin**	**Physical Activity**	-4196 (-7658 – 733.69)	**0.021**	**Physical Activity**	-	-
